# Contrasting microbial iron metabolism in sediments from oxic and hypoxic estuaries

**DOI:** 10.3389/fmicb.2026.1824768

**Published:** 2026-05-13

**Authors:** Rui Du, Cheng Xu, Duo Zhao, Hanqing Zeng, Yilin Cheng, Kai Tang, Pinghe Cai, Yao Zhang

**Affiliations:** 1 State Key Laboratory of Marine Environmental Science, College of Ocean and Earth Sciences, Xiamen University, Xiamen, China; 2 Fujian Ocean Innovation Center, Xiamen, China

**Keywords:** estuarine sediment, hypoxia, metagenomics, microbial iron metabolism, sediment core incubation

## Abstract

Estuarine sediments are pivotal zones for iron (Fe) cycling, mediated by microbial communities and coupled to carbon, nitrogen, sulfur and phosphorus transformations. However, the microbial iron metabolic processes in estuarine sediments remain poorly characterized, particularly under hypoxia. This study compared metagenomes from the Oujiang River Estuary, an oxic estuary, and the Yangtze River Estuary, a seasonally hypoxic estuary, complemented by sediment core incubations to assess geochemical responses to deoxygenation. The taxonomic affiliations of iron metabolism-related genes in the oxic estuary were homogeneous with depth, dominated by Proteobacteria and Thermodesulfobacteriota. In contrast, the hypoxic estuary exhibited strong stratification, with the surface enriched in Proteobacteria and deeper horizons dominated by Chloroflexota and *Candidatus* Bathyarchaeota. The surface sediments of the hypoxic estuary at 0–8 centimeters below the seafloor showed a hotspot with co-enrichment of dissimilatory iron reduction (e.g., *mtrABC*) and iron oxidation genes (e.g., *mtoA*) relative to both deeper layers in the same estuary and the oxic estuary, consistent with elevated genetic potential for Fe redox turnover. This hotspot also harbored high-affinity Fe acquisition systems (siderophores, inorganic Fe transporters, and heme uptake), suggesting the potential for microbial competition for iron. Co-occurrence networks connecting Fe metabolism with carbon, nitrogen, sulfur and phosphorus cycling were more complex in the hypoxic estuary than in the oxic estuary, revealing strong associations between Fe acquisition/redox cycling and organic matter turnover. A 16-day incubation of sediment cores from the oxic estuary showed that short-term deoxygenation enhanced dissolved Fe, phosphate, and ammonium release. Overall, our results suggest that bottom-water hypoxia is associated with major shifts in microbial iron metabolism potential, with implications for iron-organic matter interactions and nutrient regeneration under coastal deoxygenation.

## 1 Introduction

Estuaries represent critical transitional zones between terrestrial and marine environments, acting as highly productive ecosystems and hotspots for global biogeochemical cycling (Crump and Bowen, 2024; Kennish, 2025). Within these systems, iron (Fe) plays a pivotal dual role, serving both as an essential nutrient and a key terminal electron acceptor for microbial respiration. Although estuaries receive substantial terrestrial Fe inputs, the bioavailable fraction is regulated by rapid redox transformations and particle scavenging, particularly in oxic waters, where the poor solubility of Fe(III) constrains iron availability in marine ecosystems (Boyd and Ellwood, 2010; Norman et al., 2014; Tagliabue et al., 2017; Kappler et al., 2021). Concurrently, iron serves as a potent electron shuttle, with its redox states—ferric Fe(III) and ferrous Fe(II)—acting as key electron acceptors and donors, respectively, in a suite of microbially-mediated respiratory processes (Weber et al., 2006; Zhao et al., 2013; Zhao et al., 2015; Kappler et al., 2021). Estuarine sediments, in particular, are major reservoirs of iron (Lalonde et al., 2012; Zhao et al., 2018) and host intense microbial activity (Crump and Bowen, 2024), making them central arenas for iron transformations that profoundly influence the cycling of other major elements, including carbon (C), nitrogen (N), sulfur (S) and phosphorus (P) (Li et al., 2012).

Microorganisms are the principal drivers of sedimentary iron cycling. Dissimilatory iron-reducing bacteria (DIRB) utilize iron oxides as terminal electron acceptors for the anaerobic oxidation of organic matter, a process that mobilizes iron as soluble Fe(II) and regenerates nutrients (Li et al., 2012; Kappler et al., 2021; Zhang et al., 2021; Dong et al., 2023). In turn, the resulting Fe(II) can be re-oxidized by other microbes, such as photoferrotrophs or nitrate-reducing iron-oxidizers (Hedrich et al., 2011; Zhao et al., 2013; Zhao et al., 2015). This re-oxidation may also be promoted by cable bacteria in redox-stratified surface sediments through long-distance electron transfer (Seitaj et al., 2015; Sulu-Gambari et al., 2016a). This tight coupling of oxidative and reductive processes often leads to the formation of a “cryptic” iron cycle, where key elements are rapidly turned over within the same microenvironment (Roden, 2012; Berg et al., 2016; Nikeleit et al., 2025). Particularly in dynamic estuarine sediments, such cryptic iron cycle may be intense because traditional redox zones are highly compressed and overlapping (Burdige, 2011). The balance between the reductive and oxidative processes dictates iron’s fate—sequestration or mobilization—and is intricately linked to the availability of organic matter and the prevailing redox conditions (Dong et al., 2023).

The fact that the soluble Fe(II) mobilized by microbes is ephemeral and rapidly consumed means that its bioavailability is transient, creating a highly competitive environment which drives the evolution of diverse microbial iron acquisition strategies (Kümmerli, 2023). These mechanisms range from the direct transport of the more soluble ferrous iron across the cell membrane to more elaborate, high-affinity systems. For instance, many bacteria invest significant energy in synthesizing and secreting siderophores, small organic molecules that act as powerful chelators by scavenging Fe(III) from minerals and delivering it back to the cell via specific transporters (Kraemer et al., 2005; Wilson et al., 2016; Leventhal et al., 2019; Sheng et al., 2023; Schalk, 2025; Guo et al., 2026). Others have developed opportunistic strategies to acquire iron from organic sources, such as through the uptake and degradation of heme, an iron-containing porphyrin ring (Roe et al., 2013; Hogle et al., 2014; Hogle et al., 2017). The co-existence of these varied and often redundant systems points to intense competition for this essential resource within microbial communities (Cordero et al., 2012; Hogle et al., 2016), and as such, their distribution and regulation are highly sensitive to environmental perturbations.

In recent decades, coastal estuaries worldwide have experienced a dramatic increase in the frequency, duration, and extent of hypoxia (low dissolved oxygen, DO < 2 mg/L or ~63 μM), largely driven by anthropogenic eutrophication and climate change (Zhang et al., 2010; Dai et al., 2023). The depletion of oxygen in bottom waters acts as a powerful ecological filter and a biogeochemical switch that fundamentally alters the sedimentary environment by suppressing aerobic respiration and eliminating most oxygen-dependent benthic macrofauna, particularly bioturbators that are crucial for sediment mixing (Middelburg and Levin, 2009; Foster and Fulweiler, 2019). This leads to the enhanced preservation and accumulation of organic matter on the seafloor, forcing the resident microbial communities to shift their metabolism to anaerobic respiratory pathways (Middelburg and Levin, 2009; Jessen et al., 2017; Zhang et al., 2025). While the general effects of hypoxia on nitrogen and sulfur cycles have been extensively studied (Jäntti et al., 2021; Song et al., 2021), its specific influence on the organization of microbial iron metabolism and its interactions with other elemental cycles remains less clear. Understanding this relationship is crucial, as shifts in iron cycling can have cascading effects on carbon sequestration and nutrient regeneration.

Here, we employed a comparative metagenomic approach to analyze sediment cores from two contrasting estuaries: the oxic Oujiang River Estuary (OUE) and the seasonally hypoxic Yangtze River Estuary (YZE). We compared the differences in the taxonomic composition of microbes harboring iron metabolism genes and in the genetic potential for the iron cycle, ranging from redox transformation to diverse acquisition strategies. To understand how these processes influence other element cycles at the ecosystem level, we also compared the complexity and topology of the networks linking iron to carbon, nitrogen, sulfur, and phosphorus cycling by co-occurrence network analysis. Finally, to provide a geochemical context for iron-associated nutrient regeneration under deoxygenation, we conducted incubations of sediment cores from the oxic estuary while monitoring subsequent changes in key parameters. This study provides a framework for understanding how hypoxia correlates with microbial iron cycling in estuarine sediments and affects broader estuarine biogeochemistry.

## 2 Materials and methods

### 2.1 Sample collection

**Figure 1:**
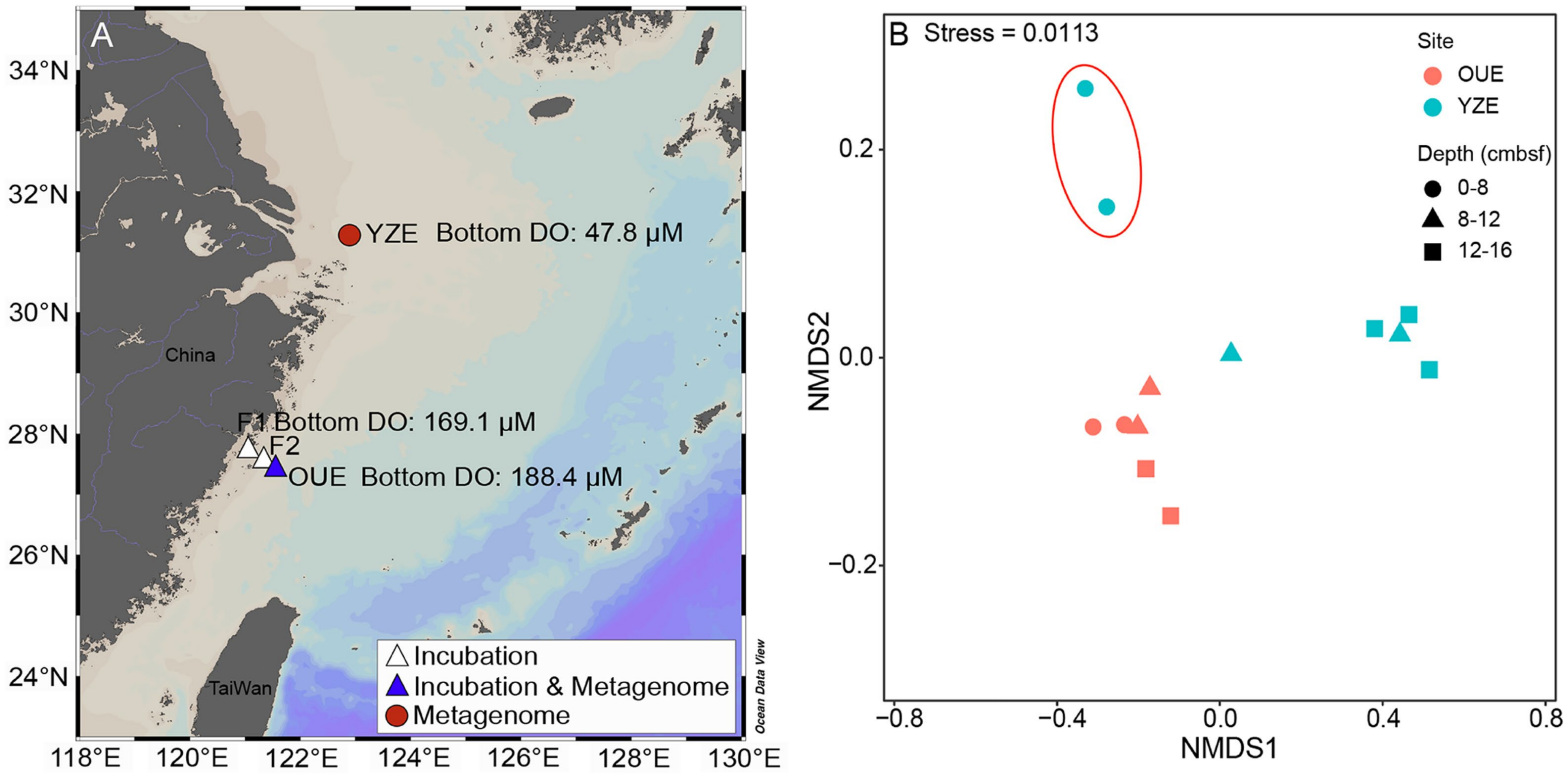
Study area and non-metric multidimensional scaling (NMDS) ordination of iron-metabolism-related taxa across sediment cores. **(A)** Map of the East China Sea with sampling locations at the Oujiang River Estuary (OUE) and Yangtze River Estuary (YZE). Bottom-water dissolved oxygen (DO, μM) during sampling is indicated for each site. **(B)** NMDS ordination based on Bray–Curtis dissimilarity calculated from Hellinger-transformed abundances of taxa affiliated with iron-metabolism genes, aggregated at the order level. Depth is reported as centimeters below the seafloor (cmbsf).

Sediment samples were collected from the OUE and YZE in the East China Sea during separate research cruises in August 2021 and 2022, respectively. Both estuaries were sampled in summer and exhibited clearly contrasting bottom-water oxygen conditions, providing a reasonable basis for comparison despite potential interannual variability. At the time of collection, the overlying bottom water at the OUE (48 m) was oxic, with dissolved oxygen (DO) concentrations of 188.4 μM, whereas the YZE (63.8 m) was characterized by hypoxia, with bottom-water DO of 47.8 μM (Figure 1A). DO concentrations were measured *in situ* using a Sea-Bird SBE 43 dissolved oxygen sensor (detection limit: 1–2 μM) (Revsbech et al., 2009) mounted on a shipboard conductivity-temperature-depth (CTD) profiler (Sea-Bird SBE 911plus). Discrete water samples collected during the cruise were analyzed via Winkler titration to verify the CTD-based measurements (Knap et al., 1996; Langdon, 2010). Sediments were collected using a box corer (20 cm×20 cm×60 cm), which was deployed vertically into the sediment to ensure the undisturbed preservation of the original stratigraphy. Sediment cores (~30 cm long) were retrieved from the box corer using a transparent PVC pipe (6.5 cm diameter). The top and bottom of each core were immediately sealed with polyethylene caps or silicone stoppers. Onboard the research vessel, the sediment cores were carefully extruded and sectioned into 4 cm intervals. Each sediment section was placed in a sterile polyethylene (PE) bag, flash-frozen in liquid nitrogen, and subsequently stored at −80 °C until DNA extraction.

### 2.2 Metagenomic sequencing, assembly, and annotation

DNA extractions from sediment samples were carried out using the FastDNA Spin Kit for Soil (MP Biomedicals) and stored at −80 °C. Metagenomic libraries were sequenced on an Illumina NovaSeq 6,000 platform (PE 2 × 150 bp, Majorbio Bio-Pharm Technology Co., Ltd., Shanghai, China). Raw paired-end sequencing reads were quality-filtered using the read_qc module in MetaWRAP (v1.3.2) (Uritskiy et al., 2018) to obtain clean reads. The filtered reads from each sample were individually assembled using MEGAHIT (v1.2.9) (Li et al., 2016) with default settings, resulting in contigs longer than 500 bp. Open reading frames (ORFs) were predicted using Prodigal (v2.6.3) (Hyatt et al., 2010) in metagenome mode (−p meta). Following this, the predicted protein sequences were clustered at 95% amino acid identity using CD-HIT (v4.8.1) (Fu et al., 2012) with parameters -c 0.95 and -aS 0.9 to generate a non-redundant prokaryotic gene database for downstream analysis. Clean metagenomic reads were mapped to the non-redundant gene catalog using BWA-MEM algorithm (Li, 2013). Gene abundance was calculated from the resulting alignments using the CoverM (v0.7.0) (Aroney et al., 2025) with following parameters: -m tpm --min-read-percent-identity 0.95 --min-read-aligned-percent 0.75 --trim-min 0.10 --trim-max 0.90. Abundances were reported as RPM (reads per million), calculated as 10^6^ × (mapped reads/gene length)/sum of (mapped reads/gene length) (Zhao et al., 2020).

The predicted genes were functionally annotated for Fe-related functions using FeGenie (Garber et al., 2020) with the hmmsearch tool. Putative gene hits were then manually curated based on the bitscore values of their HMM alignments. In parallel, the non-redundant gene set was screened against eggNOG (Huerta-Cepas et al., 2019) and Kyoto Encyclopedia of Genes and Genomes (KEGG) (Kanehisa and Goto, 2000) using DIAMOND (v2.0.11.149) (Buchfink et al., 2015) and KofamKOALA (Aramaki et al., 2020), respectively. Annotations from FeGenie, eggNOG, and KEGG were subsequently integrated through manual inspection, and genes with conflicting annotations were excluded. For the Fe-related marker genes central to this study (e.g., dissimilatory iron reduction, iron oxidation, and high-affinity iron acquisition genes), assignments were retained only when FeGenie annotations were consistent with functional annotations from eggNOG and/or KEGG, where available. This study ultimately compiled a comprehensive, high-quality database of genes implicated in Fe metabolism (Supplementary Table 1). Additionally, genes involved in major biogeochemical cycling (C, N, S and P) pathways were identified by searching against the specialized databases CCycDB (Zhou et al., 2026), NCycDB (Tu et al., 2019), SCycDB (Yu et al., 2021) and PCycDB (Zeng et al., 2022) using DIAMOND (Buchfink et al., 2015) (v2.0.11.149) with an e-value cutoff of 1 × 10^−5^. For carbon cycling, we specifically consider key microbial carbon transformation pathways covered by CCyDB: carbon fixation, respiratory carbon release, organic biosynthesis, organic degradation, organic transformation, and substrate transport (Zhou et al., 2026). Sequences were taxonomically classified using DIAMOND blastp (Buchfink et al., 2015) against the non-redundant (NR) database (NCBI, downloaded April 2023), assigning taxonomy based on the top hit with an e-value < 1 × 10^−5^.

### 2.3 Co-occurrence network analysis

Four co-occurrence networks (Fe-C, Fe-N, Fe-S, and Fe-P) were constructed using the “igraph” package in *R* for each site. For each network, pairwise Spearman’s rank correlations (*ρ*) were calculated between abundances of all Fe metabolism genes and the corresponding genes from each target elemental cycle (C, N, S, and P). An edge was considered significant if it had a correlation coefficient of |*ρ*| > 0.7 and a Benjamini-Hochberg adjusted *p*-value (FDR) < 0.01 (Liu et al., 2020). Key network properties were calculated at both the network-level (node and edge counts, graph density, average path length, modularity and diameter) and the node-level (degree, betweenness centrality and closeness centrality) to characterize each co-occurrence network. Hub genes, considered critical nodes for network topology, were identified based on their degree and betweenness centrality. We distinguished between types of hubs according to their network roles: (1) Nodes acting as major hubs within the overall network, influential both locally and globally, were defined as those with both degree and betweenness centrality exceeding the 90th percentile. (2) Nodes that serve as critical connectors or mediators linking disparate modules were additionally identified; these were defined as nodes with an exceptionally high betweenness centrality (> 90th percentile) but a non-hub level of degree (< 90th percentile) (Roth et al., 2023). All networks were visualized using the Fruchterman-Reingold layout algorithm in Gephi.^1^

Taxonomic affiliations of Fe-related hub genes in the YZE networks were derived from gene-level taxonomic annotations generated in the metagenomic annotation pipeline. For each network and sediment depth, the relative contribution of major phyla/classes to the total abundances of hub Fe genes was calculated and summarized. For module analysis, nodes were assigned to modules using the modularity optimization algorithm implemented in Gephi (Blondel et al., 2008; Bastian et al., 2009). Gene abundances were first log-transformed. Then, for each gene, the values across all sediment samples were standardized as z-scores using that gene’s own mean and standard deviation. For each sample, module abundance was calculated as the mean z-score of all genes within a given module, and these values were then summarized across sediment depths to visualize depth-associated module patterns.

To assess the robustness to node loss, we quantified the fraction of nodes remaining in the largest connected component under both random removal and targeted removal (highest-degree-first) (Tipton et al., 2018) as nodes were progressively removed. Robustness was summarized as the area under the resulting curve (AUC), where higher AUC indicates greater tolerance to node loss (Mou and Hayashi, 2025). To assess whether network contrasts could be influenced by sample variability and depth trends, we performed complementary sensitivity checks. First, we quantified beta-dispersion on Bray-Curtis dissimilarities of Hellinger-transformed gene profiles (999 permutations) to test sample variability (Anderson, 2006). Second, to reduce correlations induced by depth gradients, we reconstructed depth-controlled “residual networks.” For each gene, we regressed its relative abundance (log1p-transformed RPM) against sediment depth using a linear model and then calculated Spearman correlations on the resulting residuals (Faust, 2021). These results were reported in Supplementary Figure 3 and Supplementary Table 2.

### 2.4 Incubation experiment of sediment cores

Two sediment cores for the incubation experiment were collected from each of three sites in the oxic OUE (Figure 1A), retaining approximately 20–30 cm of the overlying water column on top of each core. Porewater was extracted from one sediment core at 2–4 cm resolution using Rhizon samplers attached to pre-cleaned 10 mL PE syringes, which had been soaked for 24 h in 0.1 M HCl and rinsed three times with ultrapure water produced by a Milli-Q Integral purification system (Merck, Darmstadt, Germany). The sediment core was sectioned at 2-cm intervals, wrapped in pre-combusted (450 °C, 4 h) aluminum foil, and archived at −20 °C for total organic carbon (TOC) and total nitrogen (TN) analysis. A separate core from each site was incubated in the dark at room temperature for 16 days. Dark incubation was used to minimize photosynthetic and photochemical effects and to focus on sediment biogeochemical responses to deoxygenation. The incubation temperature was maintained at approximately 25 °C, which falls within the range of bottom-water temperatures measured at the three OUE sites during sampling (21.8–28.1 °C). Overlying water was sampled every 24 h with a Rhizon sampler positioned 10 cm above the sediment–water interface to quantify nutrients and dissolved iron (dFe) concentrations. Upon completion of the incubation, porewater samples were similarly extracted from this core. Water samples for dFe analysis were aliquoted (≥3 mL), acidified to pH 1.6 with Optima grade HNO_3_, and stored at 4 °C until analysis. The remaining porewater/overlying water (≥10 mL) was frozen at −20 °C for subsequent nutrient analysis. The incubated sediment cores were similarly sectioned and stored for subsequent TOC and TN analysis.

Nutrient samples were measured using a Technicon AutoAnalyzer III (AA3, Bran-Lude, GmbH). Nitrate plus nitrite (N + N) was determined using copper-cadmium reduction and the pink azo dye method (Dai et al., 2008). Ammonia nitrogen was measured using the indophenol blue method (Pai et al., 2001). Soluble reactive phosphorus was determined by the phosphomolybdenum blue method (Grasshoff et al., 2009). The detection limits for N + N, ammonia nitrogen, and soluble reactive phosphorus were 0.1 μM, 0.5 μM, and 0.08 μM, respectively. For the analysis of TOC and TN, approximately 0.5 g of wet sediment was dried at 50 °C for 12 h. The dried sediment was transferred to a 50 mL acid-cleaned polypropylene centrifuge tube. Then, 5 mL of 1 mol/L HCl was added to remove inorganic carbon. The slurry was vortexed gently and allowed to react until effervescence ceased (10–15 min). Subsequently, the sample was centrifuged at 4,000 g for 10 min, and the supernatant was carefully discarded. The remaining residue was rinsed with ultrapure water, vortexed, and centrifuged again. This rinse cycle was repeated 3–5 times until the supernatant reached a neutral pH (~7). TOC and TN contents were measured using a Vario EL III elemental analyzer connected to an IsoPrime 100 isotopic ratio mass spectrometer (EA-IRMS).

The concentration of dFe in the porewater/overlying water was measured using an Inductively Coupled Plasma Mass Spectrometer (ICP-MS) following Hong et al. (2024). Briefly, samples were diluted 20-fold with 2% distilled HNO_3_ and spiked with 5 ng/g of Re, Rh, Sc and Be as internal standards. A succession of external calibration standards was prepared from certified stock solutions. Internal standard (Sc) was monitored to correct for fluctuations in Fe signal intensity. Three replicate measurements were performed, with the average deviation controlled within 5%. Procedural blanks were always below 10 nM. The dFe concentrations in the overlying water were below the method detection limit, whereas those in porewater were consistently above 2 μM. Therefore, the potential contamination during sampling and analysis is considered negligible.

### 2.5 Statistical analysis and visualization

Non-metric multidimensional scaling (NMDS) and permutational multivariate analysis of variance (PERMANOVA) were performed in *R* (vegan package) (Oksanen et al., 2025) using Bray–Curtis dissimilarities on all depth-resolved metagenomic samples (*n* = 13 in total; OUE, *n* = 6; YZE, *n* = 7). Dissimilarities were calculated from Hellinger-transformed gene abundances of taxa affiliated with iron-metabolism genes, aggregated at the order level. The differences in the relative abundance of taxa between the surface and deeper layers at YZE were examined using the linear discriminant analysis effect size (LEfSe) method. The Wilcoxon signed-rank test for paired samples was used to assess differences between the two applicable datasets from the incubation experiments. Spearman’s rank correlation was used to evaluate associations between parameters in the incubation experiments. We visualized the data using the ggplot2 (Villanueva and Chen, 2019) package in *R*.

## 3 Results and discussion

### 3.1 Contrasting stratification of iron-cycling microbes in oxic vs. hypoxic estuaries

**Figure 2:**
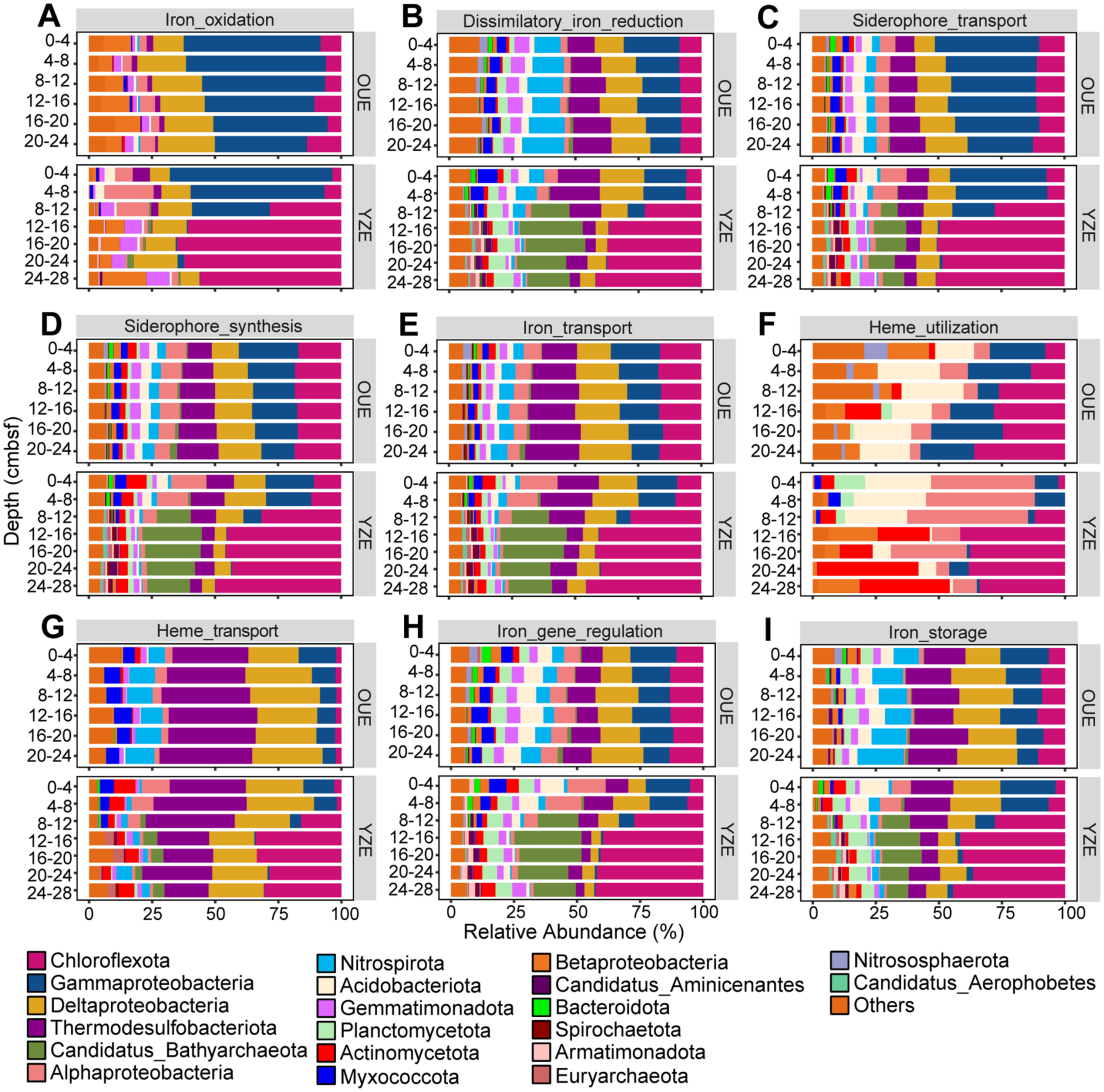
Taxonomic composition of microbial communities harboring key iron metabolism genes, stratified by sediment depth and showing the relative abundance of major phyla/classes for functional categories including **(A)** iron oxidation, **(B)** dissimilatory iron reduction (DIR), **(C)** siderophore transport, **(D)** siderophore synthesis, **(E)** iron transport, **(F)** heme utilization, **(G)** heme transport, **(H)** iron gene regulation and **(I)** iron storage.

Metagenomic analysis revealed fundamental differences in the structure of iron metabolism-associated sediment microbial communities between the oxic OUE and the hypoxic YZE. NMDS analysis of iron metabolism-related taxa at the order level (Bray–Curtis dissimilarity, Stress = 0.0113; RPM summed to orders and expressed as relative abundances; OUE, n = 6; YZE, *n* = 7) showed a statistically significant separation of microbial communities by estuary (PERMANOVA: R^2^ = 0.397, *p* = 0.011). At the OUE site, which had well-oxygenated bottom waters (188.4 μM DO), iron-metabolism-associated microbes were remarkably homogeneous across all sediment depths (Figure 1B). The taxonomic affiliations of iron-oxidation marker genes were dominated by Gammaproteobacteria from the surface (0–4 centimeters below the seafloor, cmbsf) down to 20–24 cmbsf, with their relative abundance decreasing from 54.3 to 36.6%, while Chloroflexota remained minor (≤13.5%) across all depths (Figure 2A). The composition of communities harboring dissimilatory-iron-reduction marker genes was more uniform with depth, with Gammaproteobacteria ranging from 11.9 to 22.1%, Deltaproteobacteria ranging from approximately 11.6 to 15.5%, and Thermodesulfobacteriota increasing from approximately 10.6 to 15.2% (Figure 2B). In contrast, the iron metabolism-related taxa in the hypoxic YZE (bottom-water DO: 47.8 μM) exhibited pronounced vertical stratification, segregating into distinct surface (0–8 cmbsf) and deep-layer (>8 cmbsf) clusters (Figure 1B). Both iron-oxidation and dissimilatory-iron-reduction marker genes were predominantly affiliated with Gammaproteobacteria (64.4 and 16.8%, respectively) at 0–4 cmbsf, but these affiliations dropped below 12 cmbsf. Concurrently, Chloroflexota-affiliated taxa increased to 60.8–65.0% (iron oxidation) and 5.9–41.9% (dissimilatory iron reduction) in 12–24 cmbsf layers (Figures 2A, B). In addition, the archaeal phylum *Candidatus* Bathyarchaeota contributed only 0.03% of the dissimilatory-iron-reduction associated taxa at the surface but increased markedly in deeper layers, ranging from 15.3 to 24.9% between 8 and 28 cmbsf (Figure 2B).

The relatively homogeneous OUE pattern may be related to a well-mixed estuarine setting, consistent with previous hydrodynamic studies describing the Oujiang Estuary as a macro-tidal, tide-dominated system with generally strong mixing and weak vertical density circulation (Liu et al., 2023). However, the taxonomic succession in YZE may reflect a depth-related shift in organic matter (OM) processing strategies. The Proteobacteria-dominated surface community may be associated with the turnover of relatively labile OM (Qiao et al., 2018). With increasing depth, communities were increasingly dominated by Chloroflexota and Bathyarchaeota, taxa previously associated with anaerobic processing of more complex OM (Zhou et al., 2018; Bovio-Winkler et al., 2024). These taxa may also contribute to the production of low-molecular-weight intermediates, such as acetate and H_2_, that can support anaerobic respiratory processes, including dissimilatory iron reduction (Nixon et al., 2022).

Similarly, the taxonomic composition of iron-acquisition genes (e.g., siderophore synthesis/transport, inorganic iron transport and heme transport) was vertically homogeneous in the oxic OUE but underwent a distinct taxonomic succession with depth in the YZE. In YZE surface sediments (0–8 cmbsf), taxa harboring iron-acquisition genes were dominated by Proteobacteria (e.g., Gamma-, Delta- and Alphaproteobacteria) and Thermodesulfobacteriota, a composition similar to that in OUE. However, with increasing depth, these iron-acquisition gene affiliations shifted toward Chloroflexota and *Candidatus* Bathyarchaeota (Figure 2C–E,G). The taxonomic composition of heme-utilization genes also showed distinct taxonomic stratification (Figure 2F). At the surface (0–8 cmbsf), affiliations were dominated by Alphaproteobacteria (41.2%) and Acidobacteriota (26.1%). These taxa are likely better suited to utilize heme, which is typically derived from deposited algal and biotic detritus, as a source of iron, carbon, and nitrogen (Roe et al., 2013; Hogle et al., 2014). With depth, the affiliations shifted toward Chloroflexota (41.5% at 12–16 cmbsf) and Actinomycetota (40.2% at 20–24 cmbsf), indicating a depth-related turnover in the taxa associated with heme utilization.

The taxonomic affiliations of iron regulation and storage genes also exhibited a similar vertical partitioning in YZE, pivoting from surface Proteobacteria and Thermodesulfobacteriota to Chloroflexota and *Candidatus* Bathyarchaeota (Figures 2H,I). This shift indicates a taxonomic redistribution of the genetic potential for cellular iron homeostasis, including Fur-family regulators and ferritin-superfamily storage systems (Honarmand Ebrahimi et al., 2015; Fontenot and Ding, 2023). LEfSe analysis of iron-metabolism-associated taxa statistically validated the depth-stratified community turnover in YZE (Kruskal–Wallis followed by Wilcoxon; LDA score >2.0), identifying biomarkers enriched in the upper (0–8 cmbsf; e.g., Gammaproteobacteria, Alphaproteobacteria, Myxococcales) versus deeper layers (>8 cmbsf; e.g., Anaerolineales and Dehalococcoidia from Chloroflexota, and *Candidatus* Bathyarchaeota), consistent with Figure 2 (Supplementary Figure 1).

### 3.2 The hypoxic estuary harbors a surface sediment hotspot for diverse iron cycling

**Figure 3:**
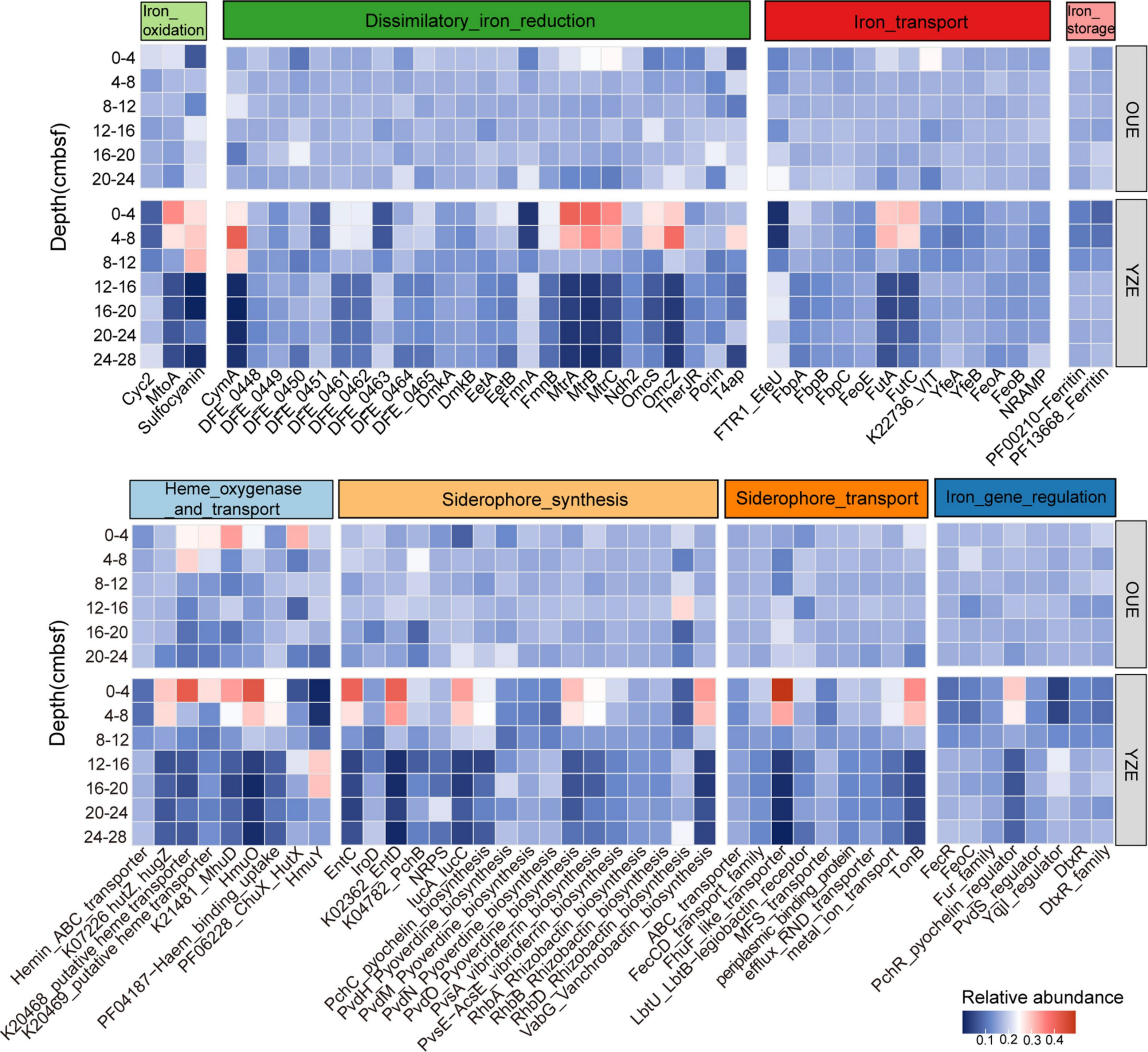
Heatmap showing the relative abundance of key iron metabolism genes across sediment depths at Oujiang River Estuary (OUE) and Yangtze River Estuary (YZE), displayed by functional category. Values are normalized for each iron-related gene within each estuary and expressed as the proportion of that gene’s total abundance (summed across all samples from the same estuary). Red, higher abundance; blue, lower abundance.

Gene relative abundance profiles showed that the YZE surface (0–8 cmbsf) was an iron metabolism hotspot where several genes for dissimilatory iron reduction and iron oxidation were enriched relative to both deeper YZE layers and the oxic OUE (Figure 3; note that the heatmap was normalized within each estuary and does not directly show absolute differences between OUE and YZE). Particularly, genes encoding extracellular electron transfer pathways for dissimilatory iron reduction were enriched in the YZE 0–8 cmbsf interval. Specifically, the Mtr complex-encoding genes (*mtrA*, *mtrB*, and *mtrC*), the outer-membrane multiheme cytochrome gene *omcS*, the periplasmic cytochrome gene *cymA*, and the accessory extracellular electron transfer genes *eetA* and *eetB* all peaked in the surface interval (0–8 cmbsf) and decreased markedly with depth. Surface abundances of these genes were 1.4–7.5-fold higher than those in deeper horizons (12–28 cmbsf), with the Mtr complex genes showing the strongest enrichment (5.0–7.5-fold), followed by *cymA* (~5.3-fold), *omcS* (~3.1-fold), and *eetA/eetB* (1.4–1.7-fold) (Figure 3). In matched-depth comparisons with OUE, the abundances of *omcS* and *eetA/eetB* in YZE (0–8 cmbsf) were 1.2–1.5 times those in OUE (0–8 cmbsf). These genes encode outer-membrane and periplasmic conduits essential for transferring electrons to extracellular solid-phase acceptors such as Fe(III) minerals (Shi et al., 2016; Light et al., 2018; Kappler et al., 2021). Their collective enrichment indicates an enhanced genetic potential for dissimilatory iron reduction in the surface sediment of YZE. For iron oxidation, the gene *mtoA* was enriched in the 0–4 cmbsf interval (2.4–6.8-fold) relative to deeper layers of the YZE. Meanwhile, a sulfocyanin-encoding gene showed 1.4 times the abundance at 0–4 cmbsf in the YZE compared to the same depth interval in the OUE. Furthermore, Cyc2, another key outer-membrane Fe(II) oxidase, showed a distinct increase with depth, with its abundance below 12 cmbsf being 1.5–2.9 times that in the YZE surface interval (0–8 cmbsf) (Figure 3). Such patterns suggest niche partitioning between outer-membrane Fe(II) oxidases, with MtoA predominant in the upper surface and Cyc2 relatively more abundant at depth. This spatial separation may reflect niche differentiation among Fe oxidation-associated microorganisms along the sediment redox gradient, potentially involving distinct oxidation pathways across depth (Laufer et al., 2016; Keffer et al., 2021; Zhou et al., 2022).

The co-enrichment of dissimilatory iron reduction and iron oxidation genes within the same sediment horizons (0–8 cmbsf) at YZE suggests an elevated genetic potential for iron redox turnover in surface sediments. In coastal sediments, such coexistence of these functional groups could be consistent with spatially coupled Fe(III) reduction and Fe(II) oxidation, potentially contributing to internal iron recycling and influencing associated organic matter and nutrient transformations (Canfield et al., 1993; Laufer et al., 2016; Sulu-Gambari et al., 2016b; Zhou et al., 2023). Such potential for coupled iron redox cycling likely makes the hypoxic YZE surface sediments a relatively dynamic transformation environment compared to the OUE system or deeper YZE layers.

In addition to redox cycling, the YZE surface also exhibited an elevated genetic potential for high-affinity iron acquisition relative to deeper YZE layers, employing at least three distinct strategies. Several genes associated with these strategies were also more abundant in YZE than in OUE. First, matched-depth comparisons showed that genes involved in siderophore biosynthesis were higher in YZE than in OUE at 0–4 cmbsf. These included pathways for catecholate siderophores (e.g., enterobactin, *entC/D*; vanchrobactin, *vabG*), hydroxamate siderophores (e.g., aerobactin, *iucA/C*), *α*-hydroxycarboxylate siderophores (e.g., vibrioferrin, *pvsE*), mixed-type pyochelin (*pchC*), as well as non-ribosomal peptide synthetase (NRPS) pathways (Figure 3). Siderophores are small, high-affinity organic chelators whose production is commonly induced when cells experience limited access to bioavailable iron, and their uptake in many Gram-negative bacteria relies on TonB-dependent outer-membrane receptors (Li et al., 2024). Correspondingly, we observed that the energy-transducing TonB system powering outer-membrane uptake via TonB-dependent receptors showed a surface maximum, with abundance in 0–4 cmbsf being ~4–5-fold higher than those in deeper horizons (12–28 cmbsf) within YZE (Figure 3). The prevalence of TonB-dependent systems may further facilitate “siderophore piracy,” whereby microbes utilize their receptors to capture siderophores produced by others, potentially intensifying iron competition (Silale and van den Berg, 2023; Schalk, 2025). Together with the elevated surface genetic potential for iron redox cycling (Figure 3), the increase in siderophore biosynthesis and TonB-dependent uptake suggests that microorganisms in YZE may still experience limited bioavailable Fe in the surface sediments, where Fe speciation, ligand binding, and mineral associations can strongly influence microbial iron availability (Gledhill and Buck, 2012; Manck et al., 2022; Guo et al., 2026).

Second, the relative abundance of genes encoding inorganic Fe(III) uptake systems also showed surface enrichment within YZE. In particular, the FutABC system, which comprises a periplasmic Fe(III)-binding component (FutA) coupled to a permease (FutB) and an ATPase (FutC) to drive high-affinity ferric iron import (Katoh et al., 2001), showed elevated abundance at 0–8 cmbsf, declining in deeper horizons (Figure 3). This indicates increased genetic potential for direct inorganic Fe(III) or weakly complexed Fe(III) acquisition near the sediment surface. In matched-depth comparisons, the FbpABC system, a periplasmic binding protein-dependent ABC transporter in which FbpA binds and shuttles Fe(III) to the inner-membrane transporter (FbpB/FbpC) for high-affinity import (Anderson et al., 2007), also showed higher *fbpA* abundance in YZE surface than in OUE at 0–4 cmbsf (~1.48 times). Together, the surface enrichment of FutABC- and FbpABC-type modules suggests that the YZE surface community is equipped with multiple siderophore-independent pathways to capture available Fe(III) in a redox-dynamic sediment.

Third, the YZE surface sediment community also displayed a genetic potential for an opportunistic acquisition strategy targeting heme as an alternative multi-element (Fe, C, N) resource (Hogle et al., 2014). This is supported by the elevated abundance of genes for heme uptake and degradation, such as the heme oxygenase gene *hmuO* (~1.81 times more abundant) and a putative heme transporter (K20468, ~5.41 times more abundant) at 0–4 cmbsf in YZE relative to OUE based on matched-depth comparisons, which would allow microbes to bypass competition for inorganic iron. Taken together, the co-enrichment of multiple iron acquisition strategies suggests the potential for microbial competition for bioavailable iron in YZE surface sediments, including siderophore production, piracy, direct inorganic iron uptake, and heme scavenging.

In line with the surface enrichment of iron redox and iron acquisition genes in YZE, we also observed a depth partitioning among iron regulatory and storage genes. The siderophore-specific regulator PchR (Ghssein and Ezzeddine, 2022) was highest at 0–4 cmbsf, whereas general iron regulators Fur family and FecR (Yokoyama et al., 2021; Kang et al., 2024) were relatively higher below the surface (Figure 3). This shift suggests that the surface community may rely more on siderophore-mediated iron acquisition, while deeper communities may depend more on general iron homeostasis systems. In parallel, ferritin-like iron storage genes were less abundant at 0–4 cmbsf and increased with depth (Figure 3). This pattern indicates an enhanced genetic potential in deeper sediments for sequestering excess ferrous iron. Such sequestration may help maintain intracellular iron homeostasis and provide an iron reservoir, while also potentially limiting iron-associated oxidative stress (Eren et al., 2024).

### 3.3 Fe-OM associations underpin microbial network complexity in the hypoxic estuary

Network analysis revealed a fundamental divergence in metabolic architecture between the two estuaries. The Fe-C, Fe-N, Fe-S and Fe-P networks in YZE all exhibited consistently higher complexity and connectivity compared to their OUE counterparts, manifested by a greater number of links, higher average degree, and increased graph density (Table 1 and Supplementary Figure 2). Concurrently, the YZE networks demonstrated greater potential efficiency, reflected in smaller network diameters and shorter average path lengths (Table 1), suggesting more rapid transfers of information or metabolites within the community network (Asif et al., 2014). Furthermore, the YZE networks displayed lower modularity relative to those in the OUE (Table 1), pointing to a less partitioned, more generalized metabolic architecture under hypoxic conditions. Robustness analysis based on simulated random and targeted node removal showed consistently higher tolerance to node loss in YZE than OUE (AUC_random = 0.312–0.392; AUC_targeted = 0.105–0.135 in YZE vs. 0.031–0.035; 0.007–0.010 in OUE) (Supplementary Figure 3), indicating greater structural redundancy in the denser YZE networks. These patterns are consistent with the idea that higher connectivity and a greater prevalence of connector/hub nodes can increase network robustness by providing alternative routes that preserve global connectivity (Lee et al., 2022; Domínguez-García and Kéfi, 2024; Naghshineh et al., 2025).

**Table 1:** Key structural properties of Fe-C, Fe-N, Fe-S and Fe-P co-occurrence networks for the oxic Oujiang River Estuary (OUE) and hypoxic Yangtze River Estuary (YZE).

Network property	Fe-C		Fe-N		Fe-S		Fe-P	
	OUE	YZE	OUE	YZE	OUE	YZE	OUE	YZE
Number of nodes	140	165	100	136	246	269	182	136
Number of links	401	1,265	216	1,066	845	3,839	516	2,031
Average node degree	5.73	15.33	4.32	15.68	6.87	28.54	5.67	39.31
Average weighted degree	5.50	14.47	4.16	14.80	6.61	27.02	5.45	37.08
Network diameter	13	9	13	10	14	10	16	7
Graph density	0.04	0.09	0.04	0.12	0.03	0.11	0.03	0.19
Modularity	0.55	0.35	0.57	0.33	0.55	0.28	0.58	0.35
Average path length	4.98	3.25	4.69	3.00	4.82	2.80	5.12	2.39

We conducted complementary sensitivity analysis to evaluate whether the observed differences in network connectivity between OUE and YZE could be explained by sample variability or depth trends. Beta-dispersion based on Bray–Curtis dissimilarities showed no significant differences in within-estuary dispersion between OUE and YZE for any of the Fe-C/N/S/P gene sets (PERMDISP, all *p* > 0.05) (Supplementary Table 2), suggesting that the network contrast is not attributable to reduced variability in OUE. Importantly, depth-controlled residual networks retained the same overall contrast: YZE remained more connected than OUE across all four networks (Supplementary Figure 4 and Supplementary Table 2).

**Figure 4:**
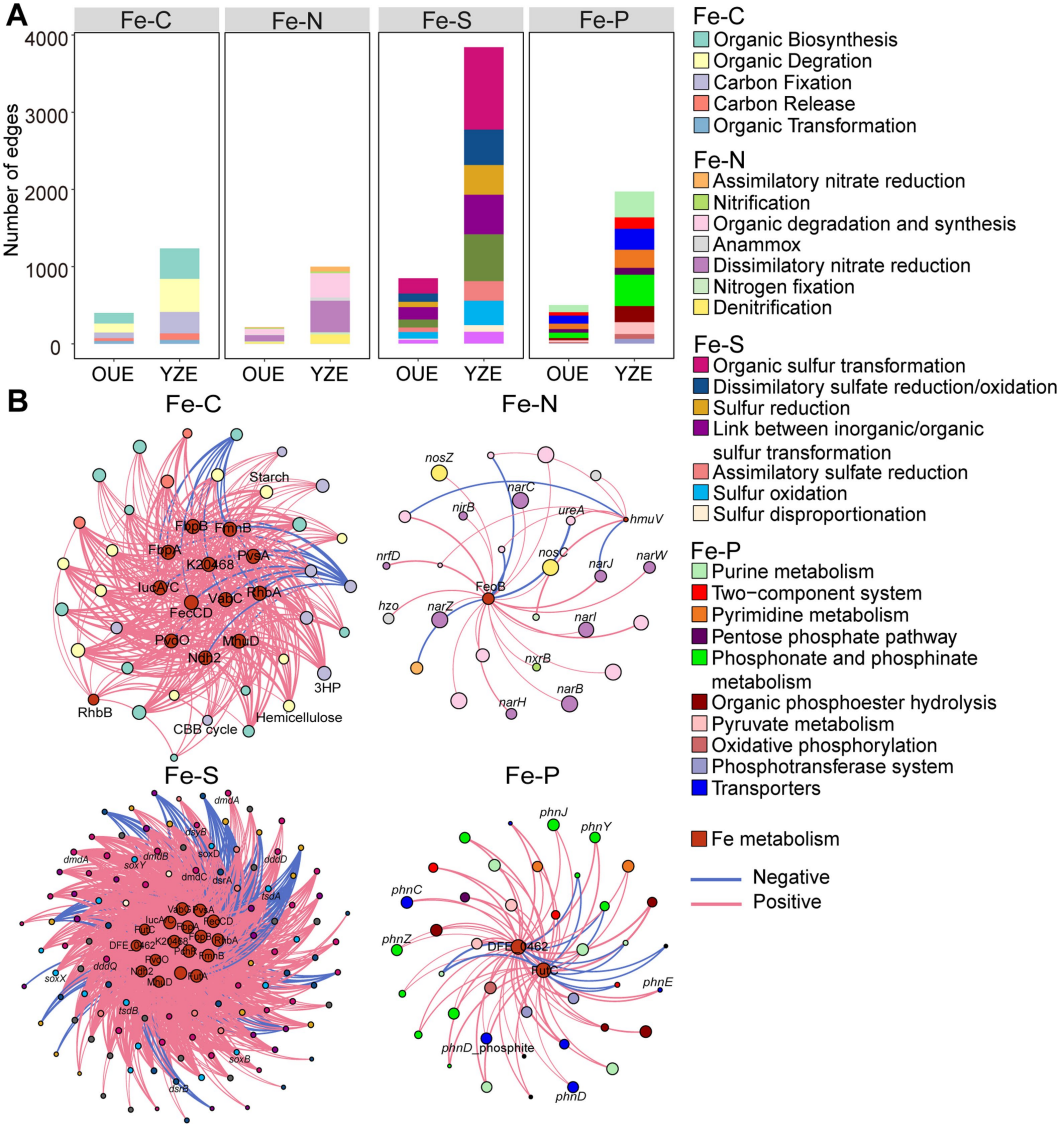
Co-occurrence networks linking iron (Fe) with carbon (C), nitrogen (N), sulfur (S) and phosphorus (P) cycling. **(A)** The number of significant correlations (edges) between Fe metabolism genes and C/N/S/P cycling pathways in the oxic Oujiang River Estuary (OUE) versus the hypoxic Yangtze River Estuary (YZE). **(B)** Simplified bipartite networks of key connections (edges) between hub iron metabolism genes (dark red nodes) and major C, N, S, and P cycling pathways (colored nodes) in the YZE. Each connection represents a strong and significant Spearman correlation (|*ρ*| > 0.7 and FDR-adjusted *p* < 0.01). Node size is proportional to its degree (number of connections). Only hub Fe metabolism genes and selected associated genes discussed in the main text are labeled.

A central finding from the network was the pronounced co-variation between iron metabolism and OM cycling. Across all networks (Fe-C, Fe-N, Fe-S and Fe-P), connectivity related to OM degradation, synthesis, and transformation was substantially enhanced in YZE relative to OUE. Most strikingly, links between iron genes and those for organic sulfur transformation increased over fivefold (from 198 in the OUE to 1,068 in the YZE), becoming the largest interaction category (Figure 4A). While these edges represent statistical co-occurrence that may partly reflect coincident responses to redox zonation, their consistent enrichment in YZE is compatible with intensified OM remineralization increasing the supply of reducing equivalents that support microbial Fe redox transformations (Dong et al., 2023).

Analysis of the YZE Fe-C network revealed that the hub genes were predominantly associated with diverse iron acquisition strategies, including siderophore synthesis (e.g., *iucA/C*, *pvsA*, *rhbA* and *pvdO*), heme utilization (K20468 and K21481), and ferric iron transport (*fbpA/B*) (Figure 4B and Supplementary Table 3). Siderophore biosynthesis genes such as *iucA/C* and *pvsA* displayed strong positive correlations with those involved in the degradation of a broad spectrum of organic substrates, including starch, hemicellulose, and aromatic compounds (Spearman’s *ρ* > 0.9, FDR-adjusted *p* < 0.01) (Figure 4B and Supplementary Table 3). These correlations likely reflect the high energy demand of siderophore production (Schalk, 2025), which in turn drives the co-enrichment of siderophore biosynthesis genes with those in carbon degradation pathways that generate energy by breaking down carbon. Moreover, genes involved in extracellular electron transfer (e.g., *ndh2*, *fmnB*) were highly positively correlated with carbon fixation pathways, including the 3-Hydroxypropionate (3-HP) bicycle and the Calvin-Benson-Bassham (CBB) cycle (Spearman’s *ρ* > 0.89, FDR-adjusted *p* < 0.01) (Figure 4B and Supplementary Table 3). These correlations suggest that extracellular electron transfer and carbon fixation pathways are co-regulated or respond similarly to the redox gradient in YZE.

The Fe-N network in YZE exhibited greater connectivity than that in OUE, which was driven by a greater number of significant correlations between iron acquisition and anaerobic nitrogen transformations (Figure 4A and Supplementary Figure 2). For instance, the ferrous iron transporter gene *feoB* showed strong positive correlations with a wide array of genes essential for dissimilatory nitrate reduction to ammonium (DNRA; *narB/C/H/I/J/W/Z*, *nirB, nrfD*), denitrification (*norC*, *nosZ*) and anammox (*hzo*) (Spearman’s *ρ* > 0.89, FDR-adjusted *p* < 0.01) (Figure 4B). These associations are consistent with the possibility that reductive dissolution elevates porewater Fe(II) in hypoxic estuarine sediments (Lau et al., 2016; Klar et al., 2017; Albertsson et al., 2019; Hird et al., 2024), creating Fe(II)-replete conditions that favor the co-selection of Fe(II) uptake and heme/Fe–S-dependent anaerobic N pathways. Conversely, *feoB* displayed strong negative correlations with genes involved in urea degradation (*ureA* and *ureC*) (Spearman’s *ρ* < −0.96, FDR-adjusted *p* < 0.01) (Figure 4B and Supplementary Table 3). This opposite pattern may reflect redox-driven niche partitioning: Fe(II)-replete, low-oxygen niches favor inorganic N respiration (e.g., DNRA, denitrification, anammox), whereas urea hydrolysis dominates in more oxygenated, bioturbated microenvironments and primarily supports nitrification (Laverock et al., 2011; Fusi et al., 2022; Arandia-Gorostidi et al., 2024).

The association between iron and sulfur metabolism constituted the largest and most complex network interaction observed in the hypoxic YZE (Table 1 and Supplementary Figure 2). Genes involved in high-affinity iron acquisition (e.g., *futA/C*, *fbpA/B, iucA/C*) contributed substantially to this complexity. They showed numerous connections to pathways for organic sulfur transformation (e.g., *tauD, dddD/Q*, and *dmdA/B/C/D*), dissimilatory sulfate reduction (e.g., *dsrA/B*), and inorganic sulfur oxidation (e.g.*, soxA/X/Y/B/D*, *sqr*, and *tsdA*) (Figure 4B and Supplementary Table 3). The dependence of key inorganic sulfur oxidation/reduction enzymes on iron-containing cofactors (e.g., heme, siroheme, Fe-S clusters, flavoproteins) provides a plausible biochemical basis for the observed connections (Ogawa et al., 2008; Ferreira et al., 2022). Consistent with this possibility, previous research has shown that some sulfate-reducing bacteria can reduce Fe(III) (Jørgensen et al., 2019). Some sulfur-oxidizing bacteria, such as *Sulfurospirillum* and *Desulfurivibrio*, can couple sulfur oxidation to Fe(III) reduction (Berg et al., 2019; Chen et al., 2025). Together, these observations support the plausibility of microbial Fe- and S-cycling interactions in hypoxic estuarine sediments, although the gene-level associations may partly reflect shared redox conditions.

The Fe-P network in the YZE presented a unique and most densely interconnected network structure, exhibiting the highest average node degree and graph density of all networks analyzed (Table 1 and Supplementary Figure 2). Functionally, the network was characterized by extensive links between iron hubs and phosphonate/phosphinate metabolism (Figure 4A and Supplementary Table 3). Strong positive correlations between the multi-heme c-type cytochrome (DFE_0462) involved in dissimilatory iron reduction and phosphonate degradation genes (e.g., *phnH*, *phnY, phnJ*) (Spearman’s *ρ* > 0.89, FDR-adjusted *p* < 0.01) (Figure 4B) suggest co-occurrence of dissimilatory iron reduction capacity with pathways that can regenerate phosphate from OM under hypoxic and potentially P-stressed conditions (Edwards et al., 2020; Amstrup et al., 2023). This pattern is consistent with the established role of dissimilatory iron reduction in benthic P cycling under hypoxia, whereby it facilitates microbial mineralization of organic P and liberates phosphate adsorbed to or co-precipitated with iron minerals (Lomnitz et al., 2016; Wang et al., 2024).

The taxonomic affiliations of hub Fe genes in the Fe-C, Fe-N, and Fe-S networks broadly followed the overall depth-dependent pattern of Fe-related taxa described above, with surface sediments mainly associated with Proteobacteria and Thermodesulfobacteriota, and deeper sediments increasingly represented by Chloroflexota and *Candidatus* Bathyarchaeota (Supplementary Figure 5). In contrast, the Fe-P network showed a markedly different taxonomic distribution. In the surface layers, hub Fe genes were mainly affiliated with Thermodesulfobacteriota, Gammaproteobacteria, and Nitrospirota, whereas deeper layers were characterized mainly by Chloroflexota together with *Candidatus* Aminicenantes and Deltaproteobacteria (Supplementary Figure 5).

To further examine module organization in the four YZE networks, we identified the modules in each network (Supplementary Figure 6). The major modules showed clear vertical partitioning along the sediment profile, with a consistent separation between surface (0–8 cmbsf) and deeper (>8 cmbsf) sediments (Supplementary Figure 7 and Supplementary Table 4). Functionally, the surface-associated modules were generally characterized by iron acquisition and redox-related functions together with OM processing. In the Fe-N and Fe-S networks, surface-associated modules were associated with nitrate-reduction and sulfur transformation functions, respectively—specifically, assimilatory sulfate reduction in the Fe-N network and sulfur reduction/oxidation-related functions in the Fe-S network (Supplementary Table 4). This vertical module partitioning was consistent with the depth-stratified distribution of Fe-related genes and taxa described above in the YZE sediments. These patterns suggest that, despite the relatively low overall modularity of the YZE networks (0.28–0.35; OUE: 0.55–0.58), their major modules still retained clear depth-associated organization.

### 3.4 Geochemical response of oxic sediments to experimentally induced deoxygenation

**Figure 5:**
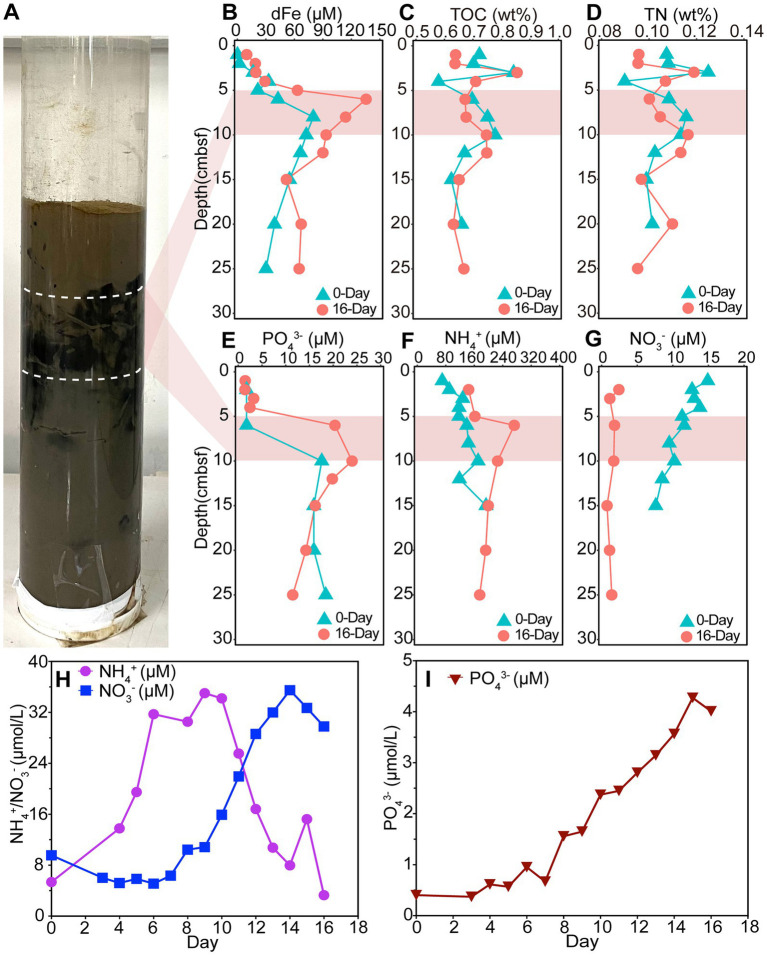
Geochemical profiles of sediments and overlying water in the Oujiang River Estuary incubation experiment. **(A)** Photograph of the sediment core after the 16-day incubation. **(B–G)** Depth profiles of dissolved iron (dFe), total organic carbon (TOC), total nitrogen (TN), phosphate (PO_4_^3−^), ammonium (NH_4_^+^) and nitrate (NO_3_^−^) concentrations (Day 0 versus Day 16) over the incubation. **(H,I)** Changes in NH_4_^+^, NO_3_^−^ and PO_4_^3−^ concentrations in the overlying water over the 16-day incubation.

To provide process-level context for the metagenomic inferences, we performed a 16-day incubation of a shallow sediment core from the OUE, collected under initially oxic bottom-water conditions (DO = 188.4 μM), which induced deoxygenation and substantial biogeochemical transformations. This was evidenced by a pronounced visual change from brown to black sediment, particularly at 5–10 cmbsf (Figure 5A), which is consistent with the reduction of iron oxide minerals and the formation of iron sulfide minerals (e.g., FeS) under anoxia (Berner, 1967; Broman et al., 2017). Specifically, dFe concentrations in the upper 12 cmbsf increased markedly from an initial 0.12–80 μM to a final range of 9.86–135.59 μM after 16 days of incubation (Wilcoxon signed-rank test, *p* < 0.01) (Figure 5B). The most substantial dFe accumulation occurred in the 5–10 cmbsf zone (Figure 5B), coinciding with the interval of solid-phase TOC and TN subtle degradation (Figures 5C,D). This observed dFe surge may indicate enhanced reductive dissolution of iron oxide minerals during deoxygenation, which could be driven by microbial dissimilatory iron reduction during the turnover of organic matter (Klar et al., 2017). While we did not quantify sulfide concentrations or pathway-specific rates in this incubation, the observed response provides geochemical context for Fe mobilization under deoxygenation. Such increases in porewater dFe are commonly observed in the shallow porewaters of organic-rich, highly productive coastal sediments under hypoxic conditions, prior to the onset of sulfidic conditions that would remove dFe via iron sulfide precipitation (Rapp et al., 2020; Wallmann et al., 2022; Yousavich et al., 2024). In contrast to the upper layers, dFe concentrations below 12 cmbsf stabilized at 52–67 μM by day 16 (Figure 5B), suggesting either limited iron reduction in the deeper anoxic zone or rapid scavenging of Fe(II) via precipitation.

Porewater phosphate (PO_4_^3−^) concentration in the upper sediment increased in parallel with dFe, notably rising from 17.6 μM (day 0) to 24.0 μM (day 16) in the 5–10 cmbsf layer (Figure 5E). The strong correlation between dFe and PO_4_^3−^ (Spearman’s *ρ* = 0.927, *p* < 0.01) suggests that phosphate, which was adsorbed on iron oxide minerals, was released upon reductive dissolution. This mechanism is a well-documented driver of phosphate regeneration in aquatic sediments, where anoxia triggers the simultaneous mobilization of iron and mineral-bound phosphate (Mortimer, 1941, 1942; Azzoni et al., 2005; Ding et al., 2016; Sun et al., 2016). This geochemical co-mobilization provides process-level context for the Fe-P associations identified in the co-occurrence network analysis (Figure 4). Mirroring the profile of dFe, PO_4_^3−^ concentrations relatively leveled off below 12 cmbsf by day 16 (Figure 5E). The incubation also revealed significant shifts in anaerobic nitrogen cycling driven by OM remineralization. In the 5–10 cmbsf zone, porewater ammonium (NH_4_^+^) concentrations increased substantially over the 16-day period, rising from an average of 153 μM to 232 μM (Figure 5F). Meanwhile, nitrate (NO_3_^−^) was almost completely consumed across the entire sediment core, dropping from 7.6–14.9 μM to 0.76–2.42 μM (Figure 5G). The substantial NH_4_^+^ accumulation alongside the depletion of NO_3_^−^ is consistent with enhanced anaerobic nitrate reduction (DNRA and/or denitrification) coupled to OM remineralization. This geochemical response provides supporting context for the frequent associations between Fe-related genes and anaerobic N-cycling genes observed in hypoxic YZE sediments in our study (Figure 4).

Nutrient flux to the overlying water during the incubation further demonstrated the sediment’s role as a net source of nutrients under induced deoxygenation. In the overlying water, NH_4_^+^ concentration increased during the first half of the incubation (day 0–10), peaking at 34.1 μM around day 10 before declining thereafter (day 10–16). In contrast, NO_3_^−^ increased steadily from 9.46 μM (day 0) to 29.7 μM (day 16) (Figure 5H). After day 10, the NO_3_^−^ trend appeared inversely related to the NH_4_^+^ decline (Spearman’s *ρ* = −0.750, *p* = 0.052), suggesting that nitrification in micro-oxic overlying water oxidized NH_4_^+^ to NO_3_^−^. This interpretation is consistent with previous research showing that nitrification can persist under low-oxygen conditions in estuarine sediments (Caffrey et al., 2019). Phosphate increased from 0.39 μM (day 0) to 4 μM (day 16) (Figure 5I). This is consistent with hypoxia-driven reductive dissolution of iron oxide minerals, which can release Fe-bound P into the porewater, followed by its subsequent diffusion across the sediment–water interface (Ding et al., 2016; Sun et al., 2016). However, dFe remained below detection limits in the overlying water throughout the incubation, despite high porewater concentrations. This absence is likely due to rapid Fe(II) oxidation at the sediment-water-interface, causing its immediate precipitation as iron oxide minerals that settle back to the sediment (Millero et al., 1987; Klar et al., 2017).

Two additional 16-day incubations of OUE sediment cores from different sites under identical conditions showed trends consistent with those described above. This included a pronounced increase in porewater dFe (Wilcoxon signed-rank test, *p* < 0.05) with concomitant PO_4_^3−^ release, NH_4_^+^ accumulation, and NO_3_^−^ drawdown. The overlying water also showed consistent trends, namely a rise in PO_4_^3−^ and complementary shifts in NH_4_^+^ and NO_3_^−^ concentrations (Supplementary Figure 8 and Supplementary Figure 9). In contrast, TOC and TN did not exhibit discernible changes over the 16 days. We posit that remineralization of a small, labile fraction of OM may drive the observed nutrient shifts without detectably altering the bulk solid-phase pool. While absolute magnitudes varied among sediment cores, trends were highly consistent, likely due to differences in reactive Fe and labile OM inventories (DeMaster et al., 2021), and other site-specific factors such as initial nitrate availability and reduced sulfur pools (Egger et al., 2016; Peng et al., 2024). Together, these incubations illustrate the potential biogeochemical consequence of short-term deoxygenation, including co-mobilization of dissolved Fe and nutrients, and provide process-level context for the associations between Fe-cycling genes and C/N/S/P-related genes observed in our metagenomic analyses.

Admittedly, a limitation of this study is the lack of matched physical and geochemical measurements for the metagenomic cores, such as porosity, permeability, grain size, sulfide, and organic matter fractions, all of which can influence oxygen penetration, solute exchange, and Fe bioavailability. The observed metagenomic contrasts between the two estuaries therefore likely reflect bottom-water hypoxia together with sediment physical-geochemical heterogeneity. In addition, because the two estuaries were sampled during separate cruises, some interannual variability may also have contributed to the observed differences. Future work integrating sediment physical characterization with in-situ redox profiling, matched geochemical measurements, and temporally synchronized sampling across estuaries will be needed to better resolve these interacting controls and to disentangle oxygen-related effects from site-specific and interannual variability.

## 4 Conclusion

This study investigated microbial iron metabolism in sediments from contrasting oxic and hypoxic estuaries using a combination of metagenomics, network analysis, and sediment core incubations. The two systems exhibited distinct community organization and functional potential. The OUE maintained relatively homogeneous communities with depth. In contrast, the YZE featured sharply stratified communities, with the surface horizon (0–8 cmbsf) serving as a key hotspot for several iron-cycling genes. This horizon was characterized by co-enriched genes associated with both dissimilatory iron reduction and iron oxidation, alongside diverse high-affinity iron acquisition systems including siderophore biosynthesis/uptake, ferric transport, and heme use. Analysis of multi-element association networks (Fe-C/N/S/P) revealed that the hypoxic YZE network was characterized by higher connectivity and lower modularity than the oxic OUE network, with hub positions frequently occupied by genes for iron acquisition and electron-transfer. These network patterns suggest stronger co-variation between iron cycling and organic matter turnover in the seasonally hypoxic YZE. Sediment incubation of OUE cores further showed that experimentally induced deoxygenation coincided with elevated porewater dFe and associated mobilization of phosphate and ammonium, providing a supporting geochemical context for the broader biogeochemical consequences of deoxygenation. Our study suggests that bottom-water hypoxia is associated with shifts in estuarine sediment communities, including enhanced surface genetic potential for iron redox turnover and iron acquisition, which may strengthen interactions between Fe and C/N/S/P processes and thereby influence nutrient regeneration. This finding has crucial implications for forecasting coastal hypoxia’s impact on carbon burial efficiency and estuarine biogeochemical stability.

## Data Availability

All data used in this study are provided in the main text and Supplementary materials. Metagenomic reads have been deposited in the National Center for Biotechnology Information (NCBI) under accession PRJNA1338721.

## Footnotes

1
https://gephi.org
