# Supplementary material for: Contrasting microbial iron metabolism in sediments from oxic and hypoxic estuaries
Source: Front Microbiol. 2026 May 13;17:1824768. doi: 10.3389/fmicb.2026.1824768 (PMC13212322; doi:10.3389/fmicb.2026.1824768)
Supplement: Supplementary file 1 [file Supplementary_file_1.DOCX]

**Supplementary Figures**


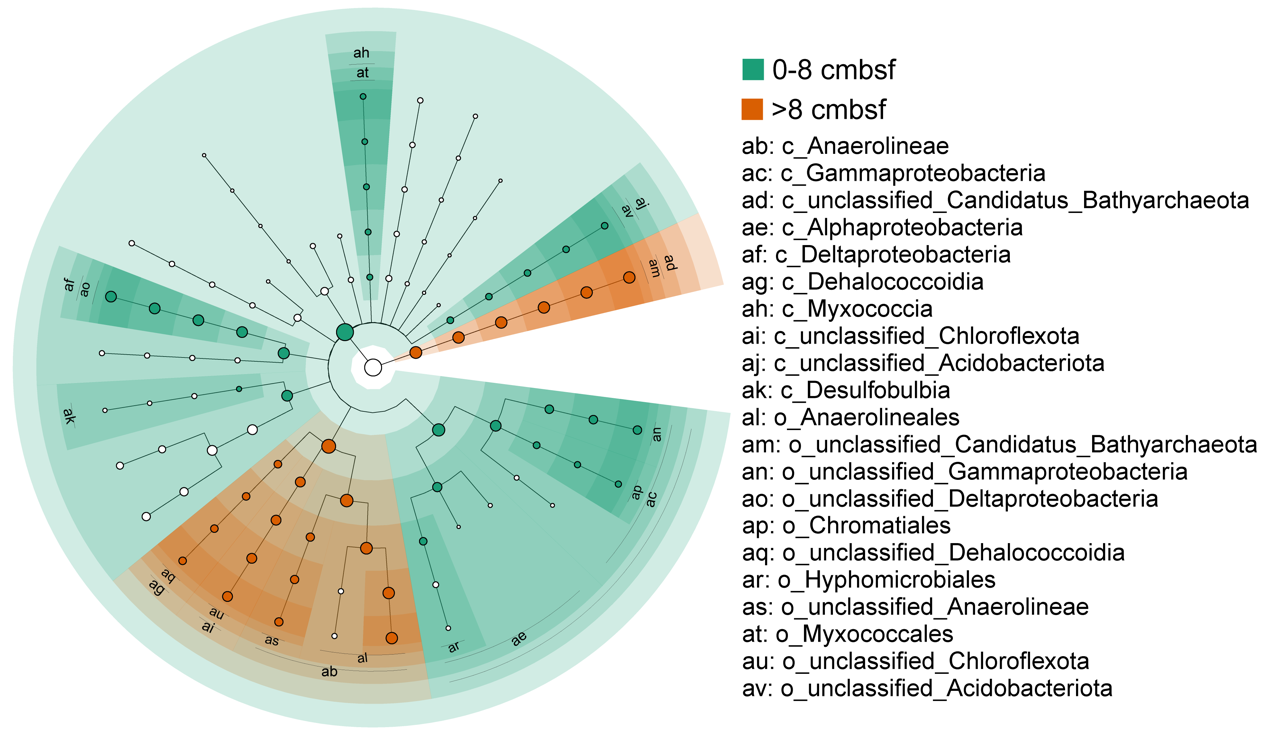


**Supplementary Figure 1** LEfSe analysis identifying differentially abundant microbial taxa between the surface (0‒8 cmbsf) and deep (>8 cmbsf) sediment layers at the hypoxic Oujiang River Estuary (YZE) site. The circles radiating from inside to outside designate the taxonomic levels from phylum to species. Each small circle represents a taxon at the corresponding taxonomic level, and its size is proportional to the relative abundance of that taxon. The colors of circles indicate the sediment layer where these taxa are significantly enriched [linear discriminant analysis (LDA) score > 2, *P* < 0.05]. White circles represent taxa with no significant enrichment between the two depth zones.


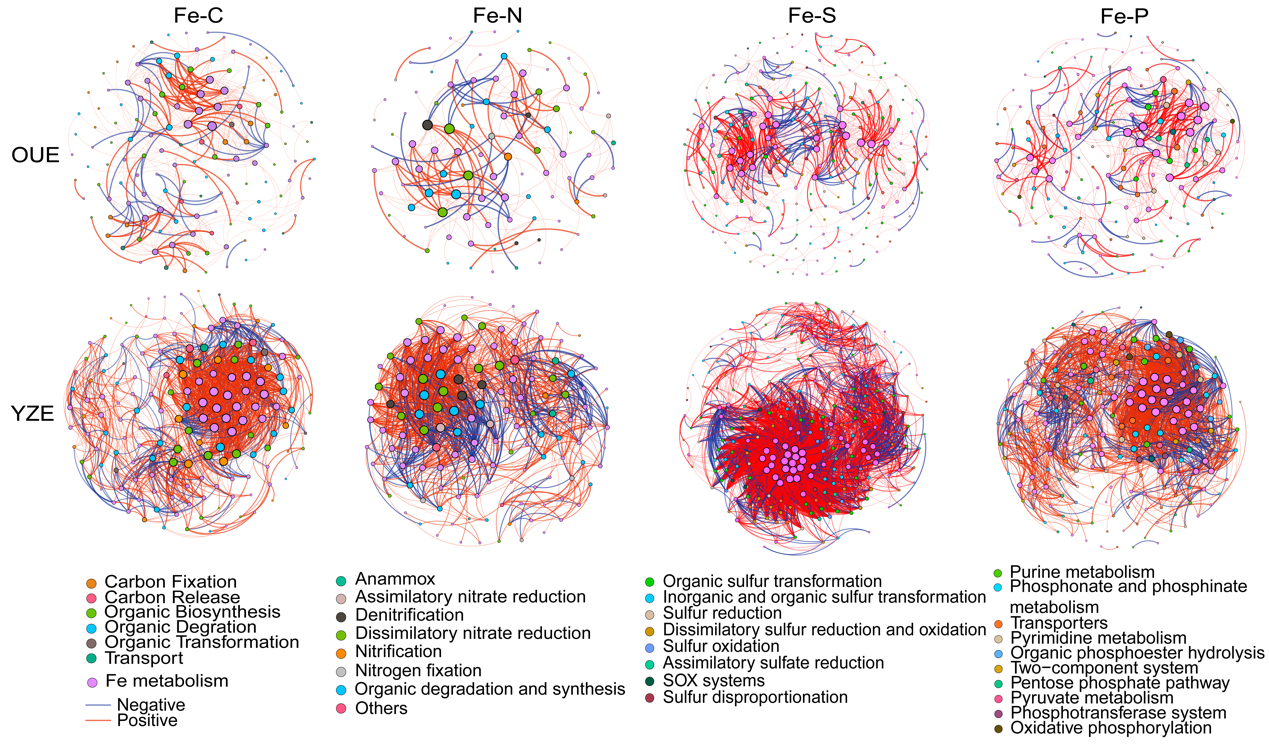


**Supplementary Figure 2** Full co-occurrence networks of iron (Fe) metabolism and carbon (C)/nitrogen (N)/sulfur (S)/phosphorus (P) cycling genes in the Oujiang River Estuary (OUE) and Yangtze River Estuary (YZE). Each connection (edge) represents a strong and significant Spearman correlation (|ρ| > 0.7 and FDR-adjusted *P* < 0.01). Nodes represent individual genes and are colored by functional pathway, with their size proportional to node degree (number of connections); edges are colored by the sign of the correlation (red for positive, blue for negative).


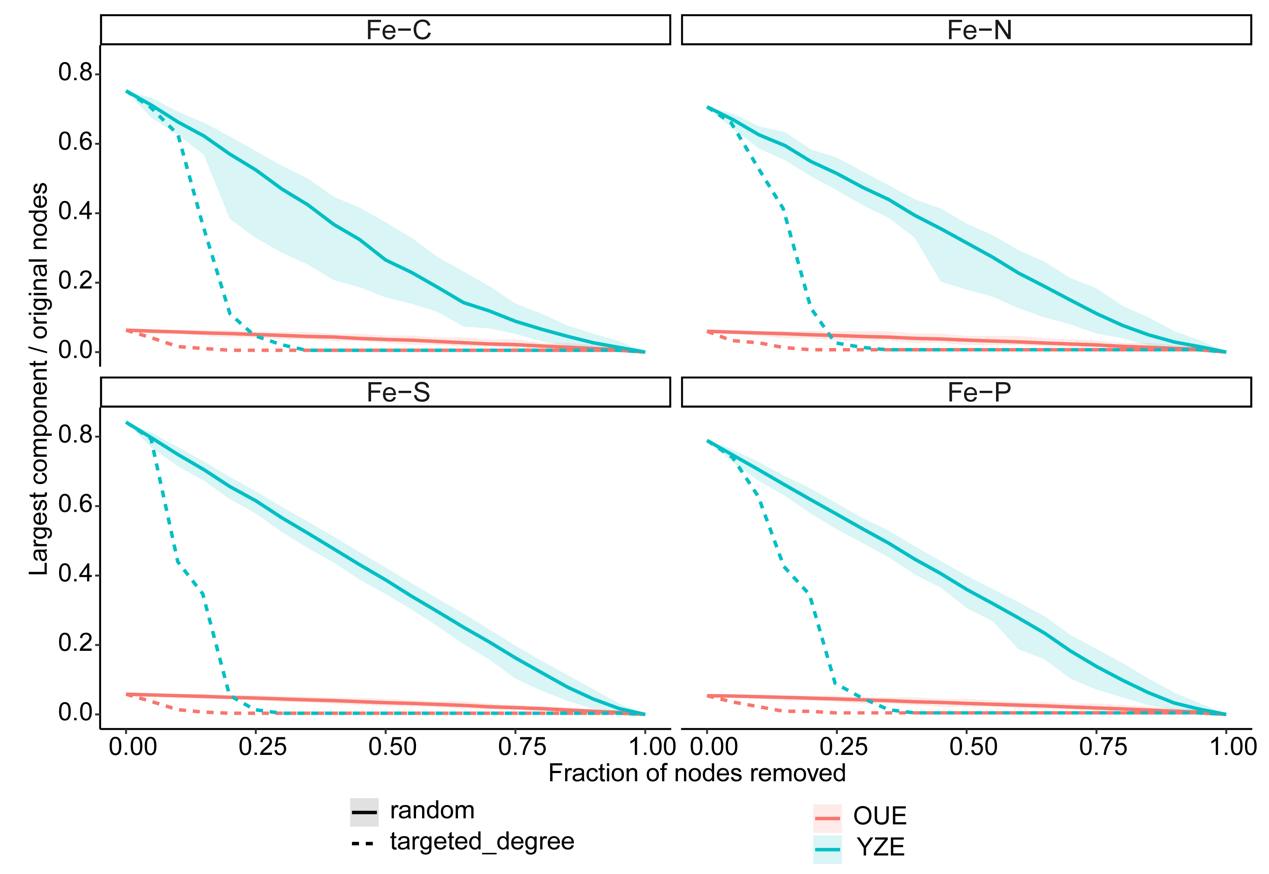


**Supplementary Figure 3** Network robustness of Fe-C, Fe-N, Fe-S and Fe-P gene co-occurrence networks in Oujiang River Estuary (OUE) and Yangtze River Estuary (YZE). Robustness was evaluated by progressively removing nodes and tracking the size of the largest connected component (LCC) relative to the original number of nodes. Curves show the mean response under random node removal (with repeated randomizations) and targeted node removal (highest-degree-first).


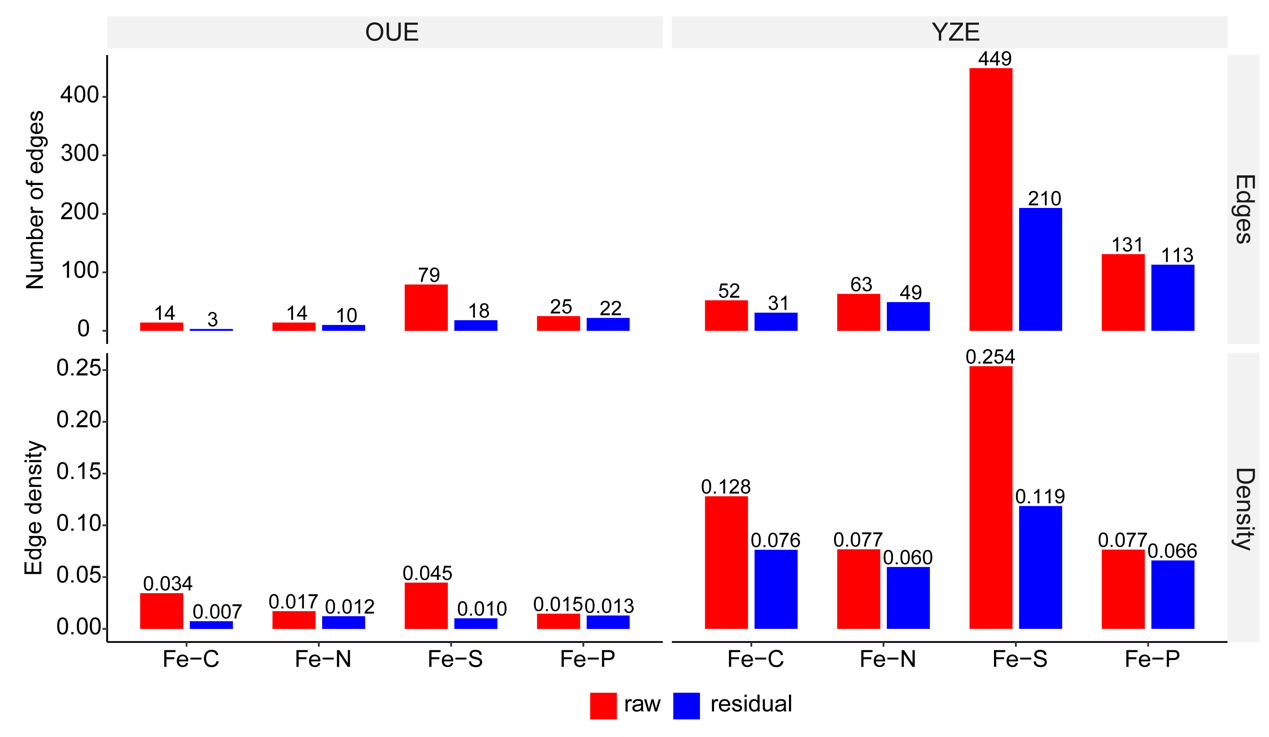


**Supplementary Figure 4** Summary of network connectivity metrics for Fe–C, Fe–N, Fe–S and Fe–P gene co-occurrence networks in Oujiang River Estuary (OUE) and Yangtze River Estuary (YZE). Bars compare raw networks (constructed from Spearman rank correlations computed directly on log1p-transformed TPM-normalized gene abundances) versus depth-controlled residual networks (constructed after regressing each gene’s log1p(TPM) against sediment depth within each estuary and computing correlations on the resulting residuals). Edges were retained if they met both a strength and significance criterion (|ρ| > 0.7 and FDR-adjusted *P* < 0.01).


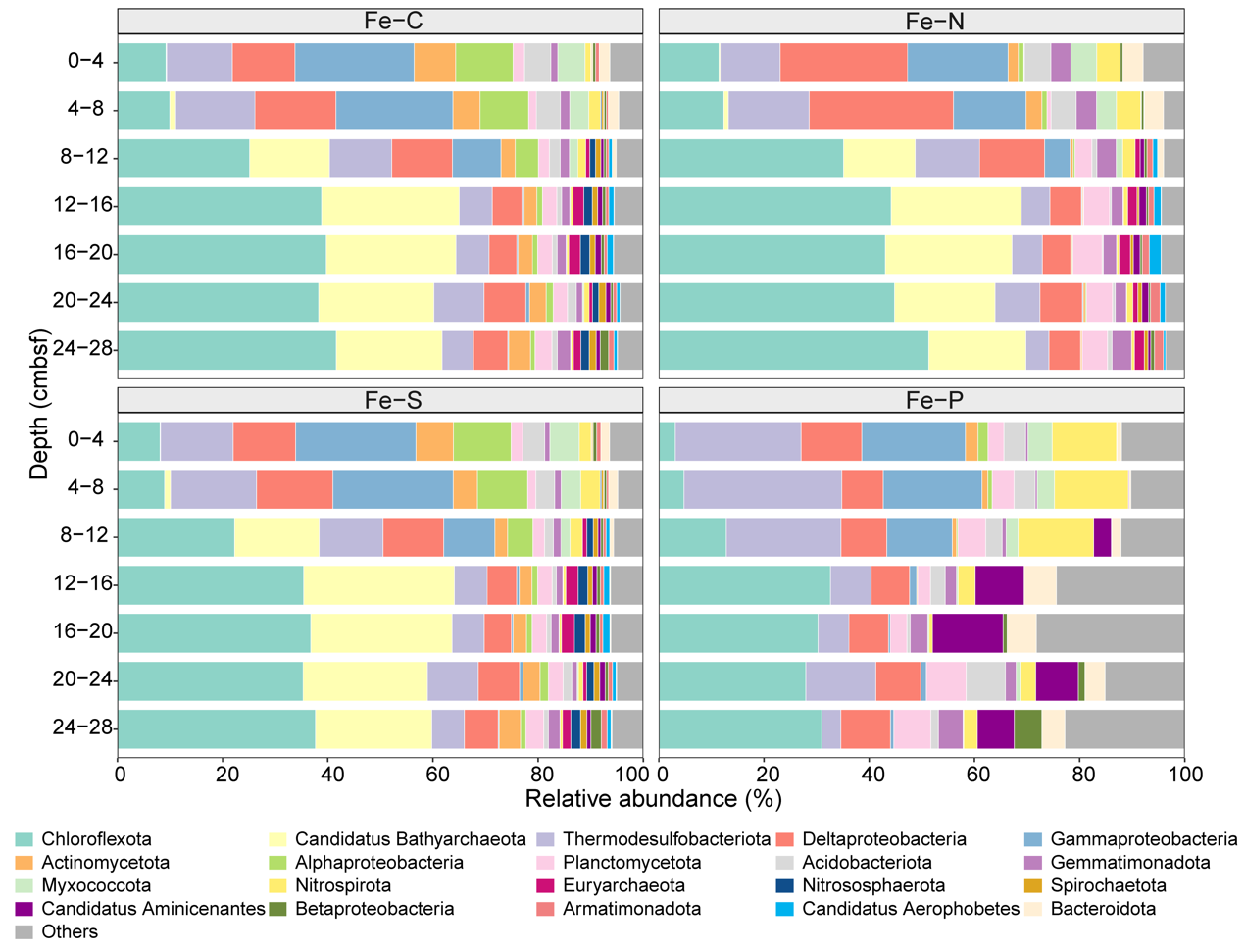


**Supplementary Figure 5** Taxonomic affiliations of hub iron (Fe) genes in the Fe-C, Fe-N, Fe-S, and Fe-P networks across sediment depths (centimeters below the seafloor, cmbsf) in the hypoxic Yangtze River Estuary. Stacked bars show the relative contribution of major phyla/classes to hub Fe gene abundance in each network.


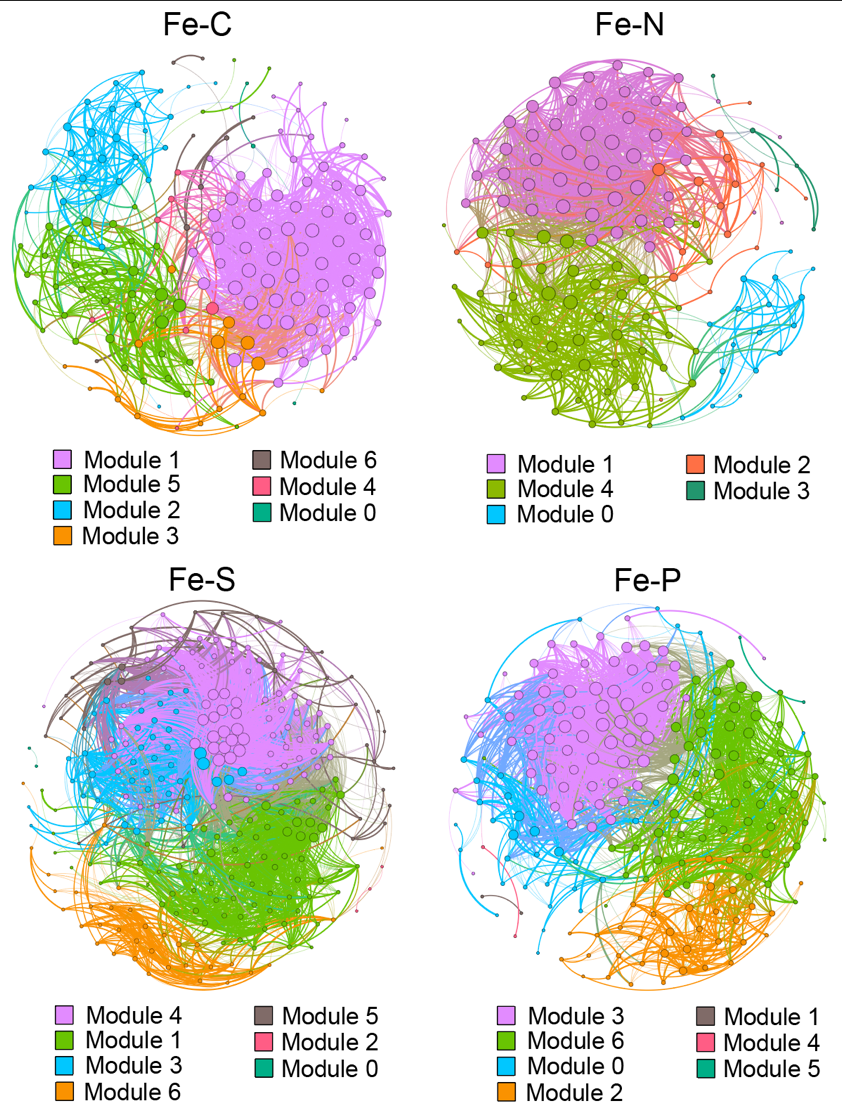


**Supplementary Figure 6** Modular organization of the Fe-C, Fe-N, Fe-S, and Fe-P co-occurrence networks in the hypoxic Yangtze River Estuary. Nodes represent genes, edges represent significant co-occurrence relationships, and colors indicate module membership.


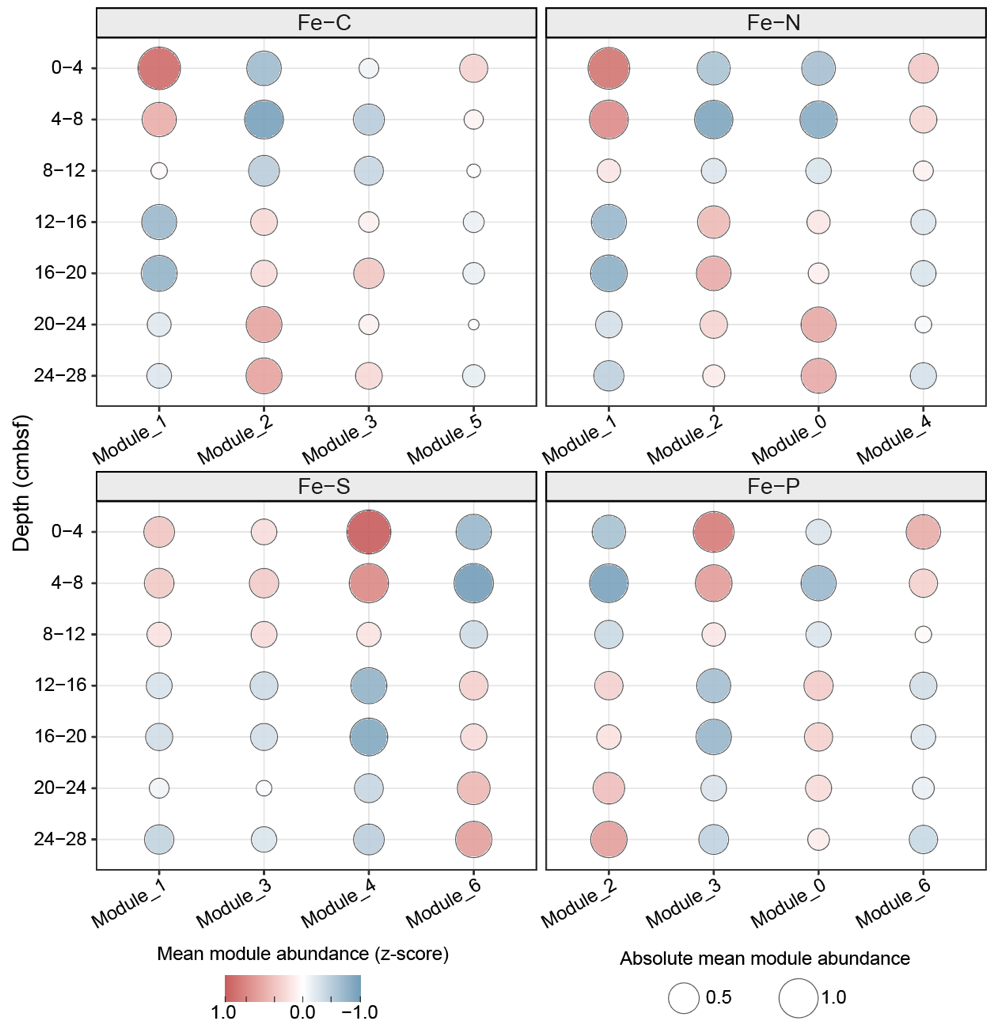


**Supplementary Figure 7** Depth distribution of major modules in the Fe-C, Fe-N, Fe-S, and Fe-P co-occurrence networks in the Yangtze River Estuary. Bubble plots show the mean abundance of the four largest modules in each network across sediment depth horizons. Bubble size indicates absolute mean module abundance, and color indicates the standardized mean abundance (z-score) of each module across depth horizons. Red indicates relatively higher abundance, and blue represents lower abundance.


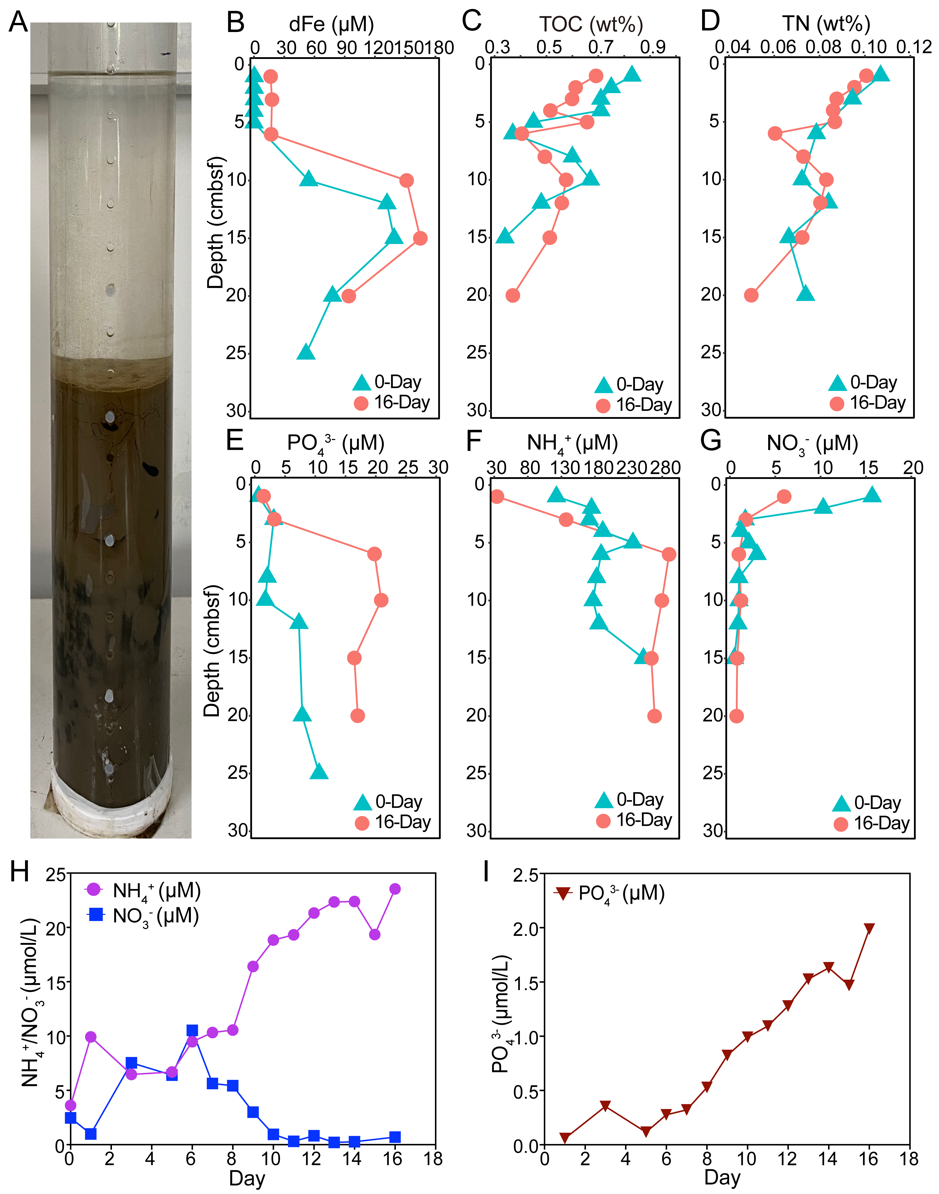


**Supplementary Figure 8** Geochemical profile of sediments and overlying water in the Oujiang River Estuary (OUE-F1) (location shown in Figure 1A) incubation experiment. (A) Photograph of the sediment core after the 16-day incubation; (B-G) Depth profiles of dissolved iron (dFe), total organic carbon (TOC), total nitrogen (TN), phosphate (PO_4_^3-^), ammonium (NH_4_^+^) and nitrate (NO_3_^-^) concentrations (Day 0 versus Day 16) over the incubation; (H-I) Changes in NH_4_^+^, NO_3_^-^ and PO_4_^3-^ concentrations in the overlying water over the 16-day incubation.


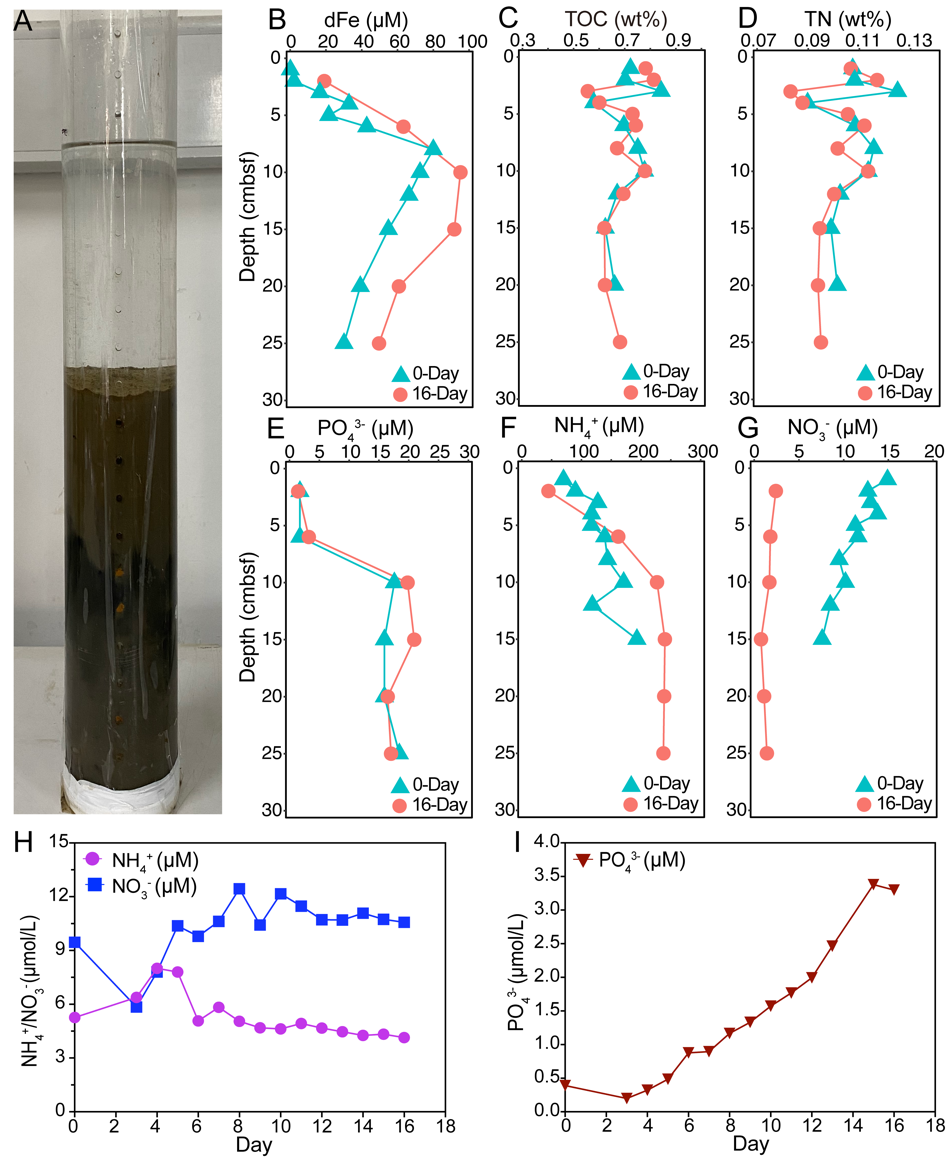


**Supplementary Figure 9** Geochemical profile of sediments and overlying water in the Oujiang River Estuary (OUE-F2) (location shown in Figure 1A) incubation experiment. (A) Photograph of the sediment core after the 16-day incubation. (B-G) Depth profiles of dissolved iron (dFe), total organic carbon (TOC), total nitrogen (TN), phosphate (PO_4_^3-^), ammonium (NH_4_^+^) and nitrate (NO_3_^-^) concentrations (Day 0 versus Day 16) over the incubation. (H-I) Changes in NH_4_^+^, NO_3_^-^ and PO_4_^3-^ concentrations in the overlying water over the 16-day incubation.
